# Innate Immunity Pathways and Breast Cancer Risk in African American and European-American Women in the Women’s Circle of Health Study (WCHS)

**DOI:** 10.1371/journal.pone.0072619

**Published:** 2013-08-21

**Authors:** Zhihong Gong, Lei Quan, Song Yao, Gary Zirpoli, Elisa V. Bandera, Michelle Roberts, Jean-Gabriel Coignet, Citadel Cabasag, Lara Sucheston, Helena Hwang, Gregory Ciupak, Warren Davis, Karen Pawlish, Lina Jandorf, Dana H. Bovbjerg, Christine B. Ambrosone, Chi-Chen Hong

**Affiliations:** 1 Roswell Park Cancer Institute, Buffalo, New York, United States of America; 2 The Cancer Institute of New Jersey, New Brunswick, New Jersey, United States of America; 3 New Jersey Department of Health, Trenton, New Jersey, United States of America; 4 Icahn School of Medicine at Mount Sinai, New York, New York, United States of America; 5 University of Pittsburgh Cancer Institute, Pittsburgh, Pennsylvania, United States of America; Health Canada and University of Ottawa, Canada

## Abstract

African American (AA) women are more likely than European American (EA) women to be diagnosed with early, aggressive breast cancer. Possible differences in innate immune pathways (e.g., inflammatory responses) have received little attention as potential mechanisms underlying this disparity. We evaluated distributions of selected genetic variants in innate immune pathways in AA and EA women, and examined their associations with breast cancer risk within the Women’s Circle of Health Study (WCHS). In stage I of the study (864 AA and 650 EA women) we found that genotype frequencies for 35 of 42 tested SNPs (18 candidate genes) differed between AAs and EAs (corroborated by ancestry informative markers). Among premenopausal AA women, comparing variant allele carriers to non-carriers, reduced breast cancer risk was associated with *CXCL5*-rs425535 (OR=0.61, *P*=0.02), while among EA women, there were associations with *TNFA*-rs1799724 (OR =2.31, *P* =0.002) and *CRP*-rs1205 (OR=0.54, *P*=0.01). For postmenopausal women, *IL1B-*rs1143627 (OR=1.80, *P*=0.02) and *IL1B-*rs16944 (OR=1.85, *P* =0.02) were associated with risk among EA women, with significant associations for *TNFA*-rs1799724 limited to estrogen receptor (ER) positive cancers (OR=2.0, *P* =0.001). However, none of the SNPs retained significance after Bonferroni adjustment for multiple testing at the level of *P*0.0012 (0.05/42) except for *TNFA*-rs1799724 in ER positive cancers. In a stage II validation (1,365 AA and 1,307 EA women), we extended evaluations for four SNPs (*CCL2*-rs4586, *CRP*-rs1205, *CXCL5*-rs425535, and *IL1RN*-rs4251961), which yielded similar results. In summary, distributions of variants in genes involved in innate immune pathways were found to differ between AA and EA populations, and showed differential associations with breast cancer according to menopausal or ER status. These results suggest that immune adaptations suited to ancestral environments may differentially influence breast cancer risk among EA and AA women.

## Introduction

Breast cancer is the most common cancer among women in the United States, accounting for 29% of all newly diagnosed cancers [1]. Although breast cancer incidence at older ages is lower among African American (AA) women than European American (EA) women, the incidence rate is higher in AA women at younger ages (50 years) [2]. More importantly, AA women are more likely to be diagnosed with aggressive tumors that are high grade and negative for estrogen receptors (ER), which are often associated with poorer disease prognosis [3]. An overrepresentation of ER negative breast cancers has also been observed in African women [4,5]. Explanations for these racial differences in breast cancer characteristics, however, are still largely unknown.

Chronic inflammation has been implicated in tumor initiation, promotion, progression, invasion, and metastasis [6]. A state of chronic inflammation develops through a complex process that involves the host’s immune system and inflammatory mediators. The innate immune system, as a dominant system of host defense, provides the first line of defense against exogenous threats such as pathogens. Activation of innate immunity promotes various inflammatory reactions and triggers the release of inflammatory cytokines as well as other inflammatory mediators, such as tumor necrosis factor alpha (*TNFα*), interleukin-1 beta (*IL1β*) and interleukin-6 (IL-6) [7]. Elevated circulating levels of inflammatory biomarkers, i.e., *IL6*, *TNFα*, C-reactive protein (CRP), have been associated with a greater risk for several types of cancer and cancer prognosis, including breast [8,9]. Several studies have also identified inflammation and immune-related signatures as being important for disease prognosis for triple negative breast cancer, the aggressive breast cancer subtype often observed in young AA women [10]. Indeed, studies have shown that AAs have higher *CRP* and *IL6* levels than EAs [11,12]. Because of evolution over millennia in Africa, and adaptation to endemic infectious diseases, it is possible that innate immune factors may differ by ancestry, with a more robust inflammatory response among AAs [13–15], which could contribute to the differential risk between AA and EA women of developing more aggressive breast cancer phenotypes.

Variants in genes involved in the innate immune response pathway may influence the production or action of inflammatory cytokines and subsequently modulate inflammatory response, influencing risk of breast cancer. Racial differences in allele frequencies of single nucleotide polymorphisms (SNPs) for certain cytokine genes have been reported in several studies, and certain genetic variants associated with increased levels of pro-inflammatory biomarkers are more frequent in AAs than that in EAs [16,17]. While a number of studies have examined inflammatory gene polymorphisms and breast cancer risk, none have focused on associations in AA women [18–21]. Furthermore, previous studies primarily focused on a small number of genes in the innate immunity pathway, most often *TNF, IL1α, IL1β, IL1* receptor antagonist (*IL1RN*), and *IL6*, with mixed results.

In this case-control study, we used a two stage design to examine potential associations between breast cancer and variants in genes involved with chronic inflammation within innate immunity-related pathways in AA and EA women. We hypothesized that the differential distribution of ‘at-risk’ alleles could contribute to the higher incidence of aggressive breast cancer among AA women, particularly risk of ER-negative breast cancers.

## Materials and Methods

### Study population

Analyses were conducted using data and samples from the Women’s Circle of Health Study (WCHS), a case-control study designed to evaluate risk factors for early/aggressive breast cancer in AA and EA women. Details of the study design, enrollment criteria, and collection of biospecimens and questionnaire data have been described previously [22,23]. In brief, cases of incident breast cancer were identified using hospital-based case ascertainment in targeted hospitals within four boroughs of the metropolitan New York City (NYC) area and by population-based rapid case ascertainment in seven counties in nearby New Jersey (NJ), through the NJ State Cancer Registry, a participant in the National Cancer Institute’s Surveillance, Epidemiology, and End Results (SEER) program. Eligible cases were English speaking women who self-identified as AA or EA, 20-75 years of age, and recently diagnosed with primary, histologically confirmed breast cancer with no previous history of cancer other than non-melanoma skin cancer. Controls were frequency matched to cases by self-reported race and 5-year age groups and were selected from the target population in the same residential area using random digit dialing supplemented by community recruitment efforts for AA women with the help of community partners and advocates. In the first stage of the study (stage I), all analyses were performed using DNA and data from 650 EA (335 cases, 315 controls) and 864 AA (458 cases, 406 controls) women. We followed up on promising findings within the entire WCHS population (stage II), with a total of 1,307 EA (658 cases, 649 controls) and 1,365 AA (621 cases, 744 controls) women.

### Ethics

This study was approved by institutional review boards at Roswell Park Cancer Institute (RPCI), the Cancer Institute of New Jersey (CINJ), Mount Sinai School of Medicine (MSSM; now the Icahn School of Medicine at Mount, Sinai), and participating hospitals in New York. Signed informed consent was obtained from each participant prior to interview and biospecimen collection.

### Data and sample collection

Detailed data on demographic characteristics, medical history, family history of cancer, and lifestyle factors were collected by in-person interviews. Anthropometric measures and biospecimens were collected by trained interviewers. Pathology data including ER status, grade and stage, and were collected and abstracted by trained study staff.

### Sample Collection and preparation

Genomic DNA was initially extracted from blood samples using the FlexiGene^TM^ DNA isolation kits (Qiagen Inc., Valencia, CA) and from Oragene^TM^ kits following the manufacturer’s protocols, but the majority of DNAs were derived from saliva samples collected using Oragene^TM^ kits (DNA Genotek Inc., Kanata, Ontario, Canada). Genomic DNA was evaluated and quantitated by Nanodrop UV-spectrometer (Thermo Fisher Scientific Inc., Wilmington, DE) and PicoGreen-based fluorometric assay (Molecular Probes, Invitrogen Inc., Carlsbad, CA), and stored at -80°C until analysis.

### SNP selection and genotyping

Forty-four SNPs were selected in eighteen candidate genes involved in innate immune response pathways by surveying the Human Genome Epidemiology (HuGE) Navigator [24]. SNPs were selected based upon their previously published associations with cancer risk and outcomes. Selected SNPs were genotyped among 864 AA and 650 EA cases and controls at the Genomics Core Facility at Roswell Park Cancer Institute using the Sequenom MassARRAY iPLEX Gold matrix-assisted laser desorption-ionization time-of-flight (MALDI-TOF) mass spectrometry assays (Sequenom Inc., San Diego, CA, US). Two SNPs were excluded from the analysis due to minor allele frequency less than 5% in AA or EA women, leaving 42 SNPs in Stage I of the study. Participant accrual continued after this initial genotyping effort and four SNPs showing suggestive associations from these initial analyses were subsequently re-genotyped in a larger WCHS sample (1,365 AA and 1,307 EA cases and controls) using the Illumina GoldenGate assay (Illumina Inc., San Diego, CA). To account for population admixture in the analysis, all samples were also genotyped for a panel of 100 ancestry informative markers (AIMs) that were previously validated in the Black Women’s Health Study [25]. Proportions of European Ancestry and African Ancestry of individual EA and AA women were computed quantitatively using the Bayesian Markov Chain Monte Carlo clustering algorithm implemented in STRUCTURE [26], based on data from the 100 genotyped AIMs. Since the sum of two ancestral proportions in each individual is always one, we used only the proportion of European Ancestry in all analyses. As a quality control measure in both genotyping efforts, five percent duplicates and two sets of in-house trio samples were included across all plates. All SNPs were in Hardy-Weinberg equilibrium (HWE) when we examined the distribution of genotypes among EA or AA controls in this study.

### Statistical Analysis

Descriptive variables were compared between cases and controls using chi-square tests for categorical variables and t-tests for continuous variables. Multivariable unconditional logistic regression was used to estimate odds ratios (ORs) and 95% confidence intervals (CIs) for the risk of breast cancer associated with genotype with adjustments for age at diagnosis (continuous), family history of breast cancer (yes, no), body mass index (continuous), education (less than or high school graduate, some college, college graduate, and graduate school), history of benign breast disease (yes, no), cigarette smoking (never smokers, former smokers, and current smokers), and proportion of European ancestry (continuous). All analyses were performed separately for EA and AA women. Participants with the most common homozygous genotype among EA controls were treated as the referent group. Co-dominant as well as dominant models (heterozygous and rare homozygous genotypes combined) were computed for all SNPs examined. Additive genotype coding based on the number of rare allele was used as ordinal variables in tests for linear trend. Analyses also were conducted to examine whether SNP associations with breast cancer risk differed by menopausal or ER status. Interactions by self-reported race were tested by including an interaction term SNP*self-reported race in multivariable logistic models. Linkage disequilibrium (LD) was determined by calculating r^2^ values between each SNP pair using the program Haploview [27].

All analyses were conducted using SAS V 9.3 (SAS Institute, Cary, CA). Statistical tests were two-sided and considered statistically significant for uncorrected *P*0.05. All significant p-values were further adjusted for multiple comparisons using Bonferroni correction, with *P*0.0012 (0.05/42) considered statistically significant.

## Results

### Participant Characteristics

Characteristics of 650 EA and 864 AA cases and controls from the initial analysis (stage I genotyping) are shown in Table 1. Among self-reported EAs and AAs, the mean proportion of European ancestry was 97-98% in EAs and 14% in AAs, respectively. EA cases were more likely than controls to have a family history of breast cancer and a history of benign breast disease, and to be less well educated. Compared to AA controls, AA cases were less likely to be current smokers, and more likely to have a history of benign breast disease. There were no other significant differences between cases and controls in either EA or AA women. Data on ER status were available for 75.8% of EA cases and 72.5% of AA cases, and AA cases were more likely than EA cases to be diagnosed with ER negative breast cancer (20.1% versus 15.5%, *P*=0.007). Characteristics of 1,307 EA and 1,365 AA cases and controls in stage II of the study, after additional participant accrual into WCHS, were generally similar to those observed among stage I participants (Table S1). However, controls were slightly younger than cases in the larger sample for both EA and AA women. Compared to AA cases, AA controls had slightly higher BMI and were more likely to be premenopausal.

**Table 1:** Characteristics of 650 European American (EA) and 864 African American (AA) cases and controls in the Women’s Health Circle of Study (WCHS)^a^.

Characteristics	European American			African American		
	Cases (n=335)	Controls (n=315)	*P*-value^c^	Cases (n=458)	Controls (n=406)	*P*-value^c^
Age (yr), mean (SD)^b^	50.5 (8.4)	50.4 (8.3)	0.90	50.9 (10.1)	50.3 (8.9)	0.36
% of European Anstry, mean (SD)	97 (9)	98 (3)	0.002	14 (16)	14 (15)	0.28
Body mass index, mean (SD)^b^	27.0 (6.3)	27.6 (7.4)	0.24	31.2 (6.8)	31.7 (7.7)	0.29
Number of full-term pregnancy	1.5 (1.3)	1.7 (1.5)	0.11	2.2 (2.0)	2.1 (1.9)	0.37
Menopausal status, n (%)			0.52			0.18
Premenopausal	186 (55.5)	167 (53.0)		242 (52.8)	196 (48.3)	
Postmenopausal	149 (44.5)	148 (47.0)		216 (47.2)	210 (51.7)	
Family history, n (%)			0.001			0.26
No	247 (73.7)	265 (84.1)		392 (85.6)	358 (88.2)	
Yes	88 (26.3)	50 (15.9)		66 (14.4)	48 (11.8)	
Education, n (%)			0.0001			0.19
Less than high school	7 (2.1)	4 (1.3)		63 (13.8)	47 (11.6)	
High school	56 (16.7)	27 (8.6)		134 (29.3)	98 (24.1)	
Some college	78 (23.3)	46 (14.6)		132 (28.8)	121 (29.8)	
College graduate	104 (31.0)	119 (37.8)		84 (18.3)	86 (21.2)	
Post-graduate degree	90 (26.9)	119 (37.8)		45 (9.8)	54 (13.3)	
History of Benign Breast Disease, n (%)			0.05			0.006
No	193 (58.7)	208 (66.2)		312 (68.4)	311 (76.8)	
Yes	136 (41.3)	106 (33.8)		144 (31.6)	94 (23.2)	
Breastfeeding, n (%)			0.31			0.97
Nulliparous	108 (32.2)	86 (27.3)		80 (17.5)	70 (17.2)	
No	72 (21.5)	66 (21.0)		170 (37.1)	154 (37.9)	
Yes	155 (46.3)	163 (51.8)		208 (45.4)	182 (44.8)	
Smoking Status, n (%)			0.40			0.005
Never Smoker	182 (54.3)	187 (59.4)		304 (66.4)	246 (60.6)	
Former Smoker	118 (35.2)	96 (30.5)		103 (22.5)	83 (20.4)	
Current Smoker	35 (10.4)	32 (10.2)		51 (11.1)	77 (19.0)	
Estrogen receptor (ER) Status						0.007
Positive	202 (60.3)			231 (50.4)		
Negative	52 (15.5)			101 (22.1)		
Unknown/missing	81 (24.2)			126 (27.5)		

^a^Number may not add up to the total number due to missing values

^b^SD: standard deviation.

^c^ *P*-value were from t-test for continuous variables and Chi-square test for categorical variables

### Relationship between genetic variants and overall breast cancer risk in AA and EA women

For 35 of the 42 SNPs, allele frequencies differed significantly between AA and EA controls (*P*0.05), and for 4 of these SNPs (*CCL2*-rs4586, *IL1B*-rs1143627, *IL1B*-rs16944, *IL8*-rs4073), the rare allele variant was reversed between the two groups. ORs, 95% CIs, and *P*-values for trend from codominant models and from dominant models between each SNP and breast cancer risk are shown in Table S2. Before stratification by menopausal or ER status, there were virtually no significant associations between any SNP examined and overall breast cancer risk in AAs or EAs except that among EA women, carriers of the *TNFA*-rs1799724 *T* allele (CT+TT) showed increased breast cancer risk (OR=1.70, 95% CI, 1.17-2.46, *P* =0.005).

### Associations stratified by menopausal status

In stratified analyses, each SNP was examined separately in pre- and post-menopausal women (Table S3). In these analyses, there were a number of SNPs that were significantly associated with breast cancer risk in either pre- or post-menopausal women, and these were not apparent in the analysis of overall risk, with results shown in Table 2.

**Table 2:** Single nucleotide polymorphisms (SNPs) of innate immune response related pathways and risk of breast cancer by menopausal status among 650.

**Gene**	**SNP**	**Genotype**	**EuropeanAmerican**						**AfricanAmerican**					
			**Pre-menopausalwomen**			**Post-menopausalwomen**			**Pre-menopausalwomen**			**Post-menopausalwomen**		
			**#Case/Control**	**OR (95%CI)^a,b^**	***P*^c,d^**	**#Case/Control**	**OR (95%CI)^a,b^**	***P*^c,d^**	**#Case/Control**	**OR (95%CI)^a,b^**	***P*^c,d^**	**#Case/Control**	**OR (95%CI)^a,b^**	***P*^c,d^**
TNFA	rs1799724	CC	118/128	1.00	0.004	97/103	1.00	0.73	224/184	1.00	0.24	204/189	1.00 (ref)	0.08
		CT	56/28	2.30 (1.31-4.04)		37/38	1.11 (0.63-1.95)		15/10	1.68 (0.7-4.02)		11/20	0.49 (0.22-1.09)	
		TT	7/4	2.38 (0.61-9.32)		3/3	1.07 (0.20-5.66)		0/0	n/a		0/0	n/a	
		CT/TT	63/32	2.31 (1.35-3.95)	0.002	40/41	1.11 (0.64-1.92)	0.71		n/a			n/a	
TNFA	rs1799964	TT	112/102	1.00	0.58	86/96	1.00	0.43	155/145	1.00	0.09	155/141	1.00	0.45
		TC	64/55	1.19 (0.72-1.94)		47/41	1.24 (0.73-2.10)		73/40	1.86 (1.17-2.95)		50/62	0.75 (0.47-1.19)	
		CC	8/6	1.06 (0.32-3.46)		7/7	1.26 (0.39-4.03)		11/10	0.95 (0.37-2.40)		10/6	1.14 (0.38-3.39)	
		TC/CC	72/61	1.17 (0.73-1.89)	0.51	54/48	1.24 (0.75-2.06)	0.40	84/50	1.67 (1.08-2.57)	0.02	60/68	0.79 (0.51-1.22)	0.29
TNFA	rs1800630	CC	133/115	1.00	0.75	100/109	1.00	0.26	179/159	1.00	0.12	168/161	1.00	0.75
		CA	46/46	0.91 (0.54-1.52)		39/35	1.24 (0.71-2.15)		54/29	1.86 (1.11-3.14)		44/47	0.91 (0.55-1.48)	
		AA	6/4	0.97 (0.23-4.11)		4/2	2.55 (0.42-15.41)		5/6	0.77 (0.22-2.69)		4/2	1.05 (0.17-6.34)	
		CA/AA	52/50	0.91 (0.55-1.50)	0.72	43/37	1.30 (0.76-2.23)	0.34	59/35	1.67 (1.02-2.72)	0.04	48/49	0.91 (0.56-1.48)	0.71
CRP	rs1205	CC	88/53	1.00	0.004	57/65	1.00	0.13	150/126	1.00	0.83	131/131	1.00	0.96
		CT	75/79	0.61 (0.37-1.01)		62/70	1.11 (0.65-1.87)		81/54	1.36 (0.88-2.11)		74/67	1.08 (0.70-1.67)	
		TT	16/26	0.34 (0.16-0.75)		17/9	2.40 (0.95-6.04)		8/14	0.43 (0.17-1.09)		10/11	0.79 (0.30-2.06)	
		CT/TT	91/105	0.54 (0.33-0.87)	0.01	79/79	1.26 (0.76-2.08)	0.36	89/68	1.16 (0.77-1.74)	0.49	84/78	1.04 (0.69-1.58)	0.85
CXCL5	rs425535	GG	132/127	1.00	0.22	113/113	1.00	0.22	95/59	1.00	0.006	73/83	1.00	0.23
		AG	41/31	1.37 (0.77-2.44)		24/32	0.69 (0.37-1.28)		116/99	0.68 (0.44-1.05)		108/99	1.28 (0.82-2.01)	
		AA	7/3	1.67 (0.37-7.41)		1/2	0.62 (0.05-7.27)		28/37	0.43 (0.23-0.80)		35/28	1.39 (0.75-2.58)	
		AG/AA	48/34	1.40 (0.81-2.43)	0.23	25/34	0.68 (0.37-1.25)	0.22	144/136	0.61 (0.40-0.93)	0.02	143/127	1.31 (0.86-2.00)	0.21
CCL2	rs4586	TT	63/78	1.00	0.04	46/63	1.00	0.77	26/31	1.00	0.05	26/14	1.00	0.87
		TC	90/64	1.71 (1.03-2.85)		78/64	1.70 (1.0-2.90)		99/92	1.26 (0.68-2.35)		90/111	0.35 (0.17-0.74)	
		CC	23/17	1.83 (0.83-4.03)		12/20	0.68 (0.28-1.64)		110/72	1.76 (0.93-3.34)		97/84	0.53 (0.25-1.13)	
		TC/CC	113/81	1.74 (1.06-2.81)	0.03	90/84	1.45 (0.87-2.41)	0.15	209/164	1.46 (0.81-2.64)	0.21	187/195	0.43 (0.21-0.88)	0.02
CCL2	rs1024611	TT	99/96	1.00	0.60	65/75	1.00	0.64	147/138	1.00	0.10	139/130	1.00	0.31
		CT	77/55	1.27 (0.78-2.05)		67/61	1.29 (0.78-2.12)		86/53	1.48 (0.96-2.28)		74/70	0.96 (0.63-1.46)	
		CC	9/12	0.93 (0.35-2.46)		9/10	0.84 (0.29-2.42)		6/4	1.29 (0.34-4.81)		3/10	0.33 (0.08-1.29)	
		CT/CC	86/67	1.21 (0.76-1.92)	0.42	76/71	1.23 (0.76-2.00)	0.51	92/57	1.47 (0.96-2.24)	0.07	77/80	0.88 (0.58-1.34)	0.56
CCL2	rs13900	CC	99/96	1.00	0.54	65/75	1.00	0.59	147/137	1.00	0.14	139/129	1.00	0.28
		CT	77/55	1.27 (0.78-2.05)		67/62	1.27 (0.77-2.10)		86/54	1.45 (0.94-2.24)		74/69	0.97 (0.63-1.48)	
		TT	9/11	0.99 (0.37-2.66)		9/9	0.92 (0.31-2.70)		5/4	1.09 (0.28-4.28)		2/10	0.25 (0.05-1.19)	
		CT/TT	86/66	1.22 (0.77-1.95)	0.39	76/71	1.23 (0.76-2.00)	0.41	91/58	1.43 (0.94-2.18)	0.10	76/79	0.89 (0.58-1.34)	0.57
IL1B	rs1143627	TT	87/74	1.00	0.71	50/71	1.00	0.003	41/33	1.00	0.87	31/41	1.00	0.67
		TC	79/71	1.06 (0.65-1.73)		62/63	1.49 (0.88-2.52)		114/86	1.11 (0.64-1.94)		104/92	1.35 (0.77-2.38)	
		CC	20/21	0.78 (0.36-1.68)		30/12	3.50 (1.55-7.94)		84/76	0.99 (0.56-1.78)		81/77	1.21 (0.67-2.18)	
		TC/CC	99/92	0.99 (0.63-1.58)	0.98	92/75	1.80 (1.09-2.95)	0.02	198/162	1.06 (0.63-1.79)	0.83	185/169	1.29 (0.76-2.19)	0.35
IL1B	rs16944	CC	88/72	1.00	0.63	49/71	1.00 (ref)	0.002	56/44	1.00	0.88	34/49	1.00	0.87
		CT	78/71	0.97 (0.60-1.58)		62/63	1.54 (0.91-2.61)		109/90	1.01 (0.61-1.66)		120/95	1.65 (0.97-2.82)	
		TT	19/20	0.80 (0.37-1.73)		30/12	3.58 (1.58-8.14)		72/61	1.04 (0.60-1.79)		59/64	1.14 (0.63-2.05)	
		CC/CT	97/91	0.93 (0.59-1.48)	0.77	92/75	1.85 (1.12-3.05)	0.02	181/151	1.02 (0.64-1.63)	0.93	179/159	1.44 (0.87-2.39)	0.16
NOD2	rs2066842	CC	100/93	1.00	0.70	84/75	1.00	0.03	210/177	1.00	0.23	190/187	1.00	0.47
		CT	71/59	0.91 (0.55-1.49)		48/62	0.60 (0.35-1.01)		28/16	1.75 (0.88-3.49)		26/22	1.36 (0.70-2.62)	
		TT	8/9	0.90 (0.32-2.57)		5/10	0.43 (0.13-1.35)		1/2	0.58 (0.05-7.26)		0/1		
		CT/TT	79/68	0.91 (0.56-1.46)	0.69	53/72	0.57 (0.35-0.96)	0.03	29/18	1.64 (0.84-3.21)	0.15	26/23	1.33 (0.69-2.57)	0.39
IL1RN	rs4251961	TT	80/63	1.00	0.99	55/52	1.00	0.22	153/133	1.00	0.57	157/133	1.00	0.04
		CT	68/72	0.76 (0.45-1.26)		70/75	0.88 (0.52-1.50)		79/57	1.16 (0.75-1.79)		51/66	0.68 (0.43-1.08)	
		CC	32/26	1.15 (0.58-2.27)		12/20	0.56 (0.24-1.30)		7/5	1.03 (0.31-3.48)		6/11	0.44 (0.15-1.31)	
		CT/CC	100/98	0.85 (0.53-1.37)	0.51	82/95	0.81 (0.49-1.35)	0.42	86/62	1.15 (0.75-1.76)	0.51	57/77	0.65 (0.42-1.01)	0.06

European American and 864 African American women in the WCHS.

^a^OR, odds ratio; 95%CI, 95% confidence interval

^b^Adjusted for age at diagnosis, education, body mass index, family history of breast cancer, history of benign breast disease, smoking status, and proportion of European ancestry.

^c^ *P-*trend for genetic dose response determined by coding genotypes as having 0, 1, or 2 variant allele, which was subsequently analyzed as an ordinal variable.

^d^ *P* for heterogeneity from dominant models (heterozygous and homozygous variant combined vs. homozygous common).

Note: *P* for interaction was for the differences in ORs between African-American and European-American women

Significant interactions were found for premenopausal women: *P* for interaction = 0.007 and 0.01 for *CXCL5*-rs425535, and *CRP*-rs1205, respectively.

Significant interactions were found for postmenopausal women: *P* for interaction = 0.04 and 0.04 for *IL1B*-rs16944, and *NOD2*-rs2066842, respectively.

Among premenopausal EA women, associations were observed for *TNFA*-rs1799724 and *CRP*-rs1205 (both *P*-trend for the T allele =0.004). Compared to the CC genotype, the combined CT and TT genotypes of *TNFA*-rs1799724 were associated with a 2.3-fold increased breast cancer risk (OR=2.31, 95% CI, 1.35-3.95), whereas carriers of the *CRP*-rs1205 T allele (CT and TT) had a 46% reduced risk (OR=0.54, 95% CI, 0.33-0.87). Among premenopausal AA women, one SNP (rs425535) in chemokine (*C-X-C* motif) ligand 5 (*CXCL5*) was observed to be inversely associated with breast cancer risk (*P*-trend for the A allele= 0.006), with AG/AA genotypes associated with a 39% reduced risk compared to the GG genotype. Suggestive associations were also observed for two *TNFA* SNPs in premenopausal AA women, with the variant TC/CC genotypes of *TNFA*-rs179964 and CA/AA genotypes of *TNFA*-rs1800630 being associated with a 1.7-fold increased breast cancer risk. Moreover, three SNPs in chemokine (C-C motif) ligand 2 (*CCL2*) (rs4586, rs1024611, rs13900) showed a suggestive non-significant elevated risk in premenopausal AA women. *CCL2*-rs4586 also was observed to be associated with an increased risk in premenopausal EA women, but the elevated risk was attenuated towards null in the stage II analysis in a larger sample of WCHS participants.

Among postmenopausal EA women, SNPs in *IL1B* (rs1143627 and rs16944) were associated with increased risk (*P*-trend for the C and T allele =0.003, 0.002, respectively) and one SNP in nucleotide-binding oligomerization domain-containing protein 2 (*NOD2*) (rs2066842) was observed to be inversely associated with risk (*P*-trend for the T allele = 0.03). The TC/CC genotypes of *IL1B*-rs1143627 or CT/TT genotypes of *IL1B*-rs16944 were associated with a 1.8-fold increased risk, whereas CT/TT genotypes of *NOD2*-rs2066842 were associated with a 43% decrease in risk. Among postmenopausal AA women, *IL1RN*-rs4251961 was inversely associated with breast cancer risk, with CT/CC genotypes associated with a decreased risk (OR=0.65, 95% CI, 0.42-1.01).

Although genotype associations with breast cancer risk differed in strength according to self-reported race, no SNP by race interactions were statistically significant except for *CXCL5*-rs425535 and *CRP*-rs1205 among premenopausal women (*P* for interaction=0.007 and 0.01, respectively), and *IL1B*-rs1143627 and *NOD2*-rs2066842 among postmenopausal women (*P* for interaction=0.04, 0.04, respectively). Of these, the interaction by race for *CXCL5*-rs425535 was most significant, where AG/AA genotypes were associated with a reduced risk among AA premenopausal women (OR=0.61, 95% CI, 0.23-0.80) and a non-significant elevated risk among EA premenopausal women (OR=1.40, 95% CI, 0.81-2.43).

### Associations stratified by ER status

Associations between each SNP and risk of ER negative and ER positive breast cancer are shown in Table S4. Although the majority of associations were similar by ER status, some did differ in stratified analyses (Table 3). Except for *TNFA* (rs179924), *NOD2* (rs2066842), and *CCL2* (rs4586), associations were distinct from those observed by menopausal status (Table 2).

**Table 3:** Single nucleotide polymorphisms (SNPs) of innate immune response related pathways and risk of breast cancer by estrogen receptor (ER) status among 650 European American and 864 African American women in the WCHS^a^.

**Gene**	**SNP**	**Genotype**	**EuropeanAmerican**						**AfricanAmerican**					
			**ERpositive**			**ERnegative**			**ERpositive**			**ERnegative**		
			**#Case/Control**	**OR (95%CI)^b,c^**	***P*^d,e^**	**#Case/Control**	**OR (95%CI)^b,c^**	***P*^d,e^**	**#Case/Control**	**OR (95%CI)^b,c^**	***P*^d,e^**	**#Case/Control**	**OR (95%CI)^b,c^**	***P*^d,e^**
TNFA	rs1799724	CC	126/231	1.00	0.001	31/231	1.00	0.11	218/373	1.00	0.53	93/373	1.00	0.68
		CT	56/66	1.88 (1.21-2.93)		16/66	1.98 (0.98-3.99)		12/30	0.80 (0.39-1.63)		7/30	1.20 (0.49-2.95)	
		TT	9/7	3.17 (1.10-9.12)		1/7	1.12 (0.12-10.58)		0/0	n/a		0/0	n/a	
		CT/TT	65/73	2.00 (1.31-3.05)	0.001	17/73	1.90 (0.96-3.77)	0.07		n/a			n/a	
FGF2	rs308379	AA	79/145	1.00	0.05	22/145	1.00	0.84	158/289	1.00	0.73	65/289	1.00	0.32
		TA	86/125	1.33 (0.88-2.00)		21/125	1.12 (0.57-2.21)		63/102	1.07 (0.73-1.58)		31/102	1.35 (0.82-2.23)	
		TT	32/39	1.70 (0.96-3.02)		6/39	1.04 (0.38-2.88)		8/12	1.08 (0.42-2.79)		3/12	1.14 (0.30-4.31)	
		TA/TT	118/164	1.41 (0.96-2.07)	0.08	27/164	1.10 (0.58-2.08)	0.76	71/114	1.07 (0.74-1.56)	0.72	34/114	1.33 (0.82-2.17)	0.25
NOD2	rs2066842	CC	104/168	1.00	0.40	35/168	1.00	0.02	200/364	1.00	0.23	88/364	1.00	0.36
		CT	74/121	0.83 (0.55-1.25)		14/121	0.45 (0.22-0.92)		31/38	1.60 (0.94-2.72)		12/38	1.56 (0.75-3.25)	
		TT	10/19	0.83 (0.36-1.93)		1/19	0.25 (0.03-1.98)		0/3			0/3		
		CT/TT	84/140	0.83 (0.56-1.23)	0.36	15/140	0.42 (0.21-0.84)	0.02	31/41	1.50 (0.89-2.54)	0.13	12/41	1.50 (0.72-3.11)	0.28
CCL5	Rs2280789	TT	140/236	1.00	0.38	37/236	1.00	0.85	136/267	1.00	0.04	71/267	1.00	0.11
		TC	56/72	1.25 (0.81-1.94)		13/72	1.23 (0.6-2.52)		80/114	1.47 (1.02-2.12)		29/114	0.89 (0.54-1.47)	
		CC	3/5	0.98 (0.21-4.54)		0/5			13/17	1.56 (0.71-3.42)		0/17		
		TC/CC	59/77	1.24 (0.81-1.89)	0.33	13/77	1.16 (0.56-2.37)	0.69	93/131	1.48 (1.04-2.11)	0.03	29/131	0.77 (0.47-1.27)	0.31
CCL5	rs2107538	CC	126/200	1.00	0.96	30/200	1.00	0.70	71/131	1.00	0.46	40/131	1.00	0.02
		CT	58/94	0.99 (0.65-1.51)		18/94	1.47 (0.75-2.87)		106/199	0.93 (0.63-1.37)		47/199	0.67 (0.41-1.11)	
		TT	6/9	0.99 (0.32-3.02)		0/9			53/73	1.24 (0.77-2.00)		13/73	0.45 (0.22-0.92)	
		CT/TT	64/103	0.99 (0.66-1.49)	0.96	18/103	1.32 (0.68-2.58)	0.41	159/272	1.01 (0.70-1.45)	0.95	60/272	0.61 (0.38-0.98)	0.04
CCL5	rs3817655	TT	128/202	1.00	0.70	32/202	1.00	0.99	73/131	1.00	0.72	41/131	1.00	0.03
		AT	54/98	0.84 (0.54-1.29)		18/98	1.24 (0.64-2.41)		105/198	0.90 (0.62-1.33)		43/198	0.62 (0.38-1.03)	
		AA	7/8	1.28 (0.43-3.82)		0/8			53/76	1.13 (0.70-1.81)		16/76	0.52 (0.27-1.02)	
		AT/AA	61/106	0.87 (0.58-1.32)	0.52	18/106	1.13 (0.58-2.19)	0.71	158/274	0.97 (0.67-1.38)	0.85	59/274	0.59 (0.37-0.95)	0.03
CCL2	rs4586	TT	64/141	1.00	0.19	19/141	1.00	0.74	21/45	1.00	0.01	15/45	1.00	0.30
		TC	99/128	1.67 (1.10-2.55)		26/128	1.46 (0.74-2.86)		96/203	1.13 (0.62-2.06)		45/203	0.65 (0.32-1.32)	
		CC	21/37	1.11 (0.57-2.15)		5/37	0.84 (0.25-2.81)		112/156	1.74 (0.95-3.20)		37/156	0.62 (0.30-1.29)	
		TC/CC	120/165	1.55 (1.03-2.31)	0.03	31/165	1.34 (0.70-2.59)	0.38	208/359	1.37 (0.77-2.44)	0.28	82/359	0.64 (0.33-1.25)	0.19

^a^Based on from 254 EA (75.8%) and 332 (72.5%) AA cases with available data on ER status.

^b^OR, odds ratio; 95%CI, 95% confidence interval

^c^Adjusted for age at diagnosis, education, body mass index, family history of breast cancer, history of benign breast disease, menopausal status, smoking status, and proportion of European ancestry.

^d^ *P-*trend for genetic dose response determined by coding genotypes as having 0, 1, or 2 variant allele, which was subsequently analyzed as an ordinal variable.

^e^ *P* for heterogeneity from dominant models (heterozygous and homozygous variant combined vs. homozygous common).

Note: *P* for interaction was for the differences in ORs between African-American and European-American women

Significant interactions were found for ER positive cancer: *P* for interaction = 0.04 for *TNFA*-rs1799724.

Significant interaction was found for ER negative cancer: *P* for interaction = 0.02 for *NOD2*-rs2066842.

Among EAs, carriers of the variant *TNFA*-rs1799724 T allele were 2-fold more likely to be diagnosed with ER positive breast cancer compared to women who were homozygous for the common allele (OR=2.0, 95% CI, 1.31-3.05, *P*=0.001), with the association remained significant after correction for multiple testing (*P*=0.04). Suggestive increased risk of ER positive cancer was also observed in EAs for carriers of *FGF2*-rs308379 TA/TT genotypes (OR=1.41, 95% CI, 0.96-2.07). Among AAs, carriers of the variant allele for *CCL5*-rs2280789 were 48% more likely to be diagnosed with ER positive breast cancer (*P*=0.03). *CCL2*-rs4586 homozygous variants (CC) were also associated with a suggestive 1.7-fold increased risk of developing ER positive cancer in AAs (OR=1.74, 95% CI, 0.95-3.20).

EA women who carry *NOD2*-rs2066842 CT/TT genotypes were observed to be 58% less likely to develop ER negative breast cancer (*P*=0.02), and among AAs, two SNPs in *CCL5* (rs2107538 and rs3817655) in LD were associated with ER negative breast cancer (*P*-trend for the T and A allele =0.02 and 0.03, respectively). Carriers of *CCL5*-rs2107538 CT/TT genotypes or *CCL5*-rs3817655 AT/AA genotypes were 40% less likely to be diagnosed with ER negative breast cancer compared to women who had CC or TT genotype, respectively. The associations for *TNFA*-rs1799724 and *NOD2*-rs2066842 were also different between AA and EA women (*P* for interaction=0.04 and 0.02, respectively), with a significant increased risk of ER positive or decreased risk of ER negative breast cancer in EA, but not in AA women.

### Stage II genotyping in larger WCHS population

We extended analysis for four SNPs, *CCL2*-rs4586, *CRP*-rs1205, *CXCL5*-rs425535, *IL1RN*-rs4251961, to a larger dataset after accrual of an additional 501 AA and 657 EA cases and controls into the WCHS. Associations by menopausal status and by ER status are presented in Tables 4 and 5. The results, overall, were very similar in direction and magnitude to results obtained with the smaller Stage I participant pool, except that the borderline elevated risk (OR=1.74, 95% CI, 1.06-2.81) associated with *CCL2*-rs4586 TC/CC genotypes in premenopausal EA women was attenuated to the null in the larger dataset (OR=0.94, 95% CI, 0.68-1.29). In addition, we observed a significant interaction between AA and EA women for the association of *CCL2*-rs4586 with risk of ER positive cancers in the larger data set (*P* for interaction=0.04). In comparison with results from the smaller population, a stronger significantly increased risk (OR=1.95, 95% CI, 1.20-2.60) was observed for AA women who carried the *CCL2*-rs4586 CC genotype compared to those with TT genotypes.

**Table 4:** Four Single nucleotide polymorphisms (SNPs) of innate immune response related pathways replicated among 1,307 European American and 1,365 African American women in the WCHS: with risk of breast cancer by menopausal status.

**Gene**	**SNP**	**Genotype**	**EuropeanAmerican**						**AfricanAmerican**					
			**Pre-menopausalwomen**			**Post-menopausalwomen**			**Pre-menopausalwomen**			**Post-menopausalwomen**		
			**#Case/Control**	**OR (95%CI)^a,b^**	***P*^c,d^**	**#Case/Control**	**OR (95%CI)^a,b^**	***P*^c,d^**	**#Case/Control**	**OR (95%CI)^a,b^**	***P*^c,d^**	**#Case/Control**	**OR (95%CI)^a,b^**	***P*^c,d^**
CCL2	rs4586	TT	133/143	1.00	0.70	113/120	1.00	0.92	33/58	1.00	0.02	31/35	1.00	0.56
		TC	162/166	0.94 (0.67-1.32)		154/129	1.38 (0.94-2.01)		126/196	1.14 (0.69-1.89)		131/150	0.86 (0.49-1.52)	
		CC	47/45	0.92 (0.56-1.53)		38/43	0.83 (0.47-1.44)		147/157	1.63 (0.98-2.71)		150/145	1.03 (0.58-1.81)	
		TC/CC	209/211	0.94 (0.68-1.29)	0.70	192/172	1.23 (0.86-1.77)	0.26	273/353	1.35 (0.84-2.18)	0.22	281/295	0.94 (0.55-1.62)	0.83
CRP	rs1205	CC	152/127	1.00	0.02	134/126	1.00	0.68	194/279	1.00	0.38	189/218	1.00	0.43
		CT	148/172	0.72 (0.51-1.00)		142/126	1.16 (0.80-1.68)		100/108	1.46 (1.04-2.07)		112/99	1.33 (0.94-1.89)	
		TT	43/55	0.62 (0.38-1.02)		31/34	1.02 (0.57-1.82)		12/24	0.72 (0.34-1.51)		11/15	0.74 (0.32-1.75)	
		CT/TT	191/227	0.69 (0.50-0.95)	0.02	173/160	1.13 (0.79-1.60)	0.50	112/132	1.32 (0.95-1.84)	0.10	123/114	1.25 (0.89-1.76)	0.19
CXCL5	rs425535	GG	253/285	1.00	0.04	238/221	1.00	0.39	118/135	1.00	0.06	109/125	1.00	0.18
		AG	79/62	1.51 (1.02-2.24)		64/67	0.81 (0.53-1.24)		150/206	0.84 (0.60-1.18)		155/167	1.10 (0.77-1.58)	
		AA	10/7	1.53 (0.54-4.32)		5/4	0.95 (0.23-3.89)		38/70	0.62 (0.38-1.01)		48/40	1.46 (0.87-2.45)	
		AG/AA	89/69	1.51 (1.04-2.20)	0.03	69/71	0.82 (0.54-1.24)	0.34	188/276	0.78 (0.57-1.08)	0.14	203/207	1.17 (0.83-1.65)	0.36
IL1RN	rs4251961	TT	147/137	1.00	0.56	130/107	1.00	0.28	202/277	1.00	0.99	224/219	1.00	0.18
		CT	130/165	0.78 (0.55-1.10)		136/143	0.83 (0.57-1.22)		95/123	0.99 (0.70-1.39)		77/100	0.77 (0.53-1.11)	
		CC	66/52	1.32 (0.84-2.08)		40/42	0.78 (0.45-1.34)		9/10	1.06 (0.40-2.77)		11/13	0.79 (0.33-1.89)	
		CT/CC	196/217	0.91 (0.66-1.25)	0.56	176/185	0.82 (0.57-1.18)	0.28	104/133	1.00 (0.71-1.39)	0.98	88/113	0.77 (0.54-1.10)	0.15

^a^OR, odds ratio; 95%CI, 95% confidence interval

^b^Models were Adjusted for age at diagnosis, education, body mass index, family history of breast cancer, history of benign breast disease, smoking status, and the proportion of European ancestry.

^c^ *P-*trend for genetic dose response determined by coding genotypes as having 0, 1, or 2 variant allele, which was subsequently analyzed as an ordinal variable.

^d^ *P* for heterogeneity from dominant models (heterozygous and homozygous variant combined vs. homozygous common).

Note: *P* for interaction was for the differences in ORs between African-American and European-American women:

Significant interactions were found for premenopausal women: *P* for interaction = 0.02 and 0.005 for *CRP*-rs1205 and *CXCL5*-rs425535, respectively.

**Table 5:** Four Single nucleotide polymorphisms (SNPs) of innate immune response related pathways replicated among 1,307 European American and 1,365 African American women in the WCHS: with risk of breast cancer by estrogen receptor (ER) status^a^.

**Gene**	**SNP**	**Genotype**	**EuropeanAmerican**						**AfricanAmerican**					
			**ERpositive**			**ERnegative**			**ERpositive**			**ERnegative**		
			**#Case/Control**	**OR (95%CI)^b,c^**	***P*^d,e^**	**#Case/Control**	**OR (95%CI)^b,c^**	***P*^d,e^**	**#Case/Control**	**OR (95%CI)^b,c^**	***P*^d,e^**	**#Case/Control**	**OR (95%CI)^b,c^**	***P*^d,e^**
CCL2	rs4586	TT	147/263	1.00	0.40	31/263	1.00	0.77	27/93	1.00	0.002	17/93	1.00	0.79
		TC	189/295	1.08 (0.81-1.44)		40/295	1.14 (0.68-1.9)		135/346	1.38 (0.85-2.24)		67/346	1.02 (0.56-1.86)	
		CC	43/88	0.74 (0.47-1.15)		9/88	0.78 (0.35-1.75)		161/302	1.95 (1.20-3.18)		65/302	1.07 (0.59-1.95)	
		TC/CC	232/383	1.00 (0.76-1.31)	0.98	49/383	1.05 (0.64-1.73)	0.84	296/648	1.63 (1.02-2.60)	0.04	132/648	1.04 (0.59-1.84)	0.88
CRP	rs1205	CC	170/259	1.00	0.14	39/259	1.00	0.57	204/497	1.00	0.54	91/497	1.00	0.48
		CT	169/298	0.83 (0.62-1.10)		29/298	0.67 (0.40-1.14)		105/207	1.26 (0.93-1.69)		54/207	1.47 (1.00-2.15)	
		TT	43/89	0.77 (0.50-1.18)		12/89	1.01 (0.50-2.05)		14/39	0.80 (0.42-1.55)		4/39	0.56 (0.19-1.64)	
		CT/TT	212/387	0.81 (0.62-1.07)	0.13	41/387	0.75 (0.46-1.21)	0.24	119/246	1.18 (0.89-1.58)	0.24	58/246	1.32 (0.91-1.92)	0.14
CXCL5	rs425535	GG	285/506	1.00	0.28	63/506	1.00	0.67	126/260	1.00	0.31	52/260	1.00	0.90
		AG	86/129	1.15 (0.83-1.59)		17/129	1.05 (0.58-1.91)		155/373	0.87 (0.65-1.16)		76/373	1.03 (0.69-1.53)	
		AA	10/11	1.44 (0.58-3.58)		0/11			42/110	0.83 (0.54-1.28)		21/110	0.94 (0.53-1.66)	
		AG/AA	96/140	1.17 (0.86-1.60)	0.32	17/140	0.96 (0.53-1.74)	0.90	197/483	0.86 (0.65-1.14)	0.29	97/483	1.01 (0.69-1.48)	0.96
IL1RN	rs4251961	TT	155/244	1.00	0.96	34/244	1.00	0.70	227/496	1.00	0.29	95/496	1.00	0.32
		CT	162/308	0.88 (0.66-1.18)		33/308	0.76 (0.45-1.28)		84/223	0.79 (0.58-1.08)		49/223	1.22 (0.82-1.81)	
		CC	64/94	1.09 (0.73-1.62)		13/94	0.99 (0.49-1.99)		12/23	1.04 (0.49-2.19)		5/23	1.25 (0.45-3.48)	
		CT/CC	226/402	0.93 (0.71-1.22)	0.60	46/402	0.81 (0.50-1.32)	0.40	96/246	0.82 (0.61-1.10)	0.18	54/246	1.22 (0.83-1.79)	0.30

^a^Based on data from 468 EA (71.1%) and 473 (76.2%) AA cases with available data on ER status.

^b^OR, odds ratio; 95%CI, 95% confidence interval

^c^Models were Adjusted for age at diagnosis, education, body mass index, family history of breast cancer, history of benign breast disease, menopausal status, smoking status, and the proportion of European ancestry.

^d^ *P-*trend for genetic dose response determined by coding genotypes as having 0, 1, or 2 variant allele, which was subsequently analyzed as an ordinal variable.

^e^ *P* for heterogeneity from dominant models (heterozygous and homozygous variant combined vs. homozygous common).

Note: *P* for interaction was for the differences in ORs between African-American and European-American women:

Significant interaction was found for ER positive cancer: *P* for interaction = 0.04 for *CCL2*-rs4586.

## Discussion

Recently, a number of genome-wide association (GWA) studies focused on breast cancer have been completed, and have identified novel genetic variants as potentially being associated with breast cancer risk [28]. GWA studies are able to screen a large number of SNPs covering the whole genome, but it may not always be the most optimal approach to detect certain important variants and newly discovered genetic variants may only explain a small fraction of population risk [29,30]. Evaluating functional gene variants in candidate pathways is an important hypothesis driven complementary method for increasing our knowledge of potentially important biological pathways in breast cancer risk. In this case-control study, we comprehensively examined common genetic variants within innate immunity pathways with overall risk of breast cancer, as well as breast cancer risk by menopausal and ER status in AA and EA women. We found that genotype frequencies for 35 out of 42 SNPs were significantly different between AA and EA women, with only one SNP, *TNFA*-rs1799724, being significantly associated with overall breast cancer risk among EA women. SNP associations with breast cancer risk, however, were found to vary substantially between AA and EA populations when menopausal or ER status was considered. Our findings suggest that different gene networks may be associated with breast cancer in AA versus EA women, pre- versus post-menopausal women, and in ER positive versus ER negative breast cancers, and provide insights into the etiology of breast cancer within these subgroups, indicating areas for further research into reasons for early onset/aggressive breast cancer in AA women.

The potential impact of inflammation-related susceptibility loci that are unevenly distributed within populations as a contributor to observed heterogeneity in breast cancer phenotypes and risk between different racial groups has been understudied. As reviewed by Pennington and colleagues [31] and Chapman and Hill [13] the high burden of infectious diseases in tropical Africa and the pressure to survive such life-threatening illnesses likely led to selection for those with more robust innate immune responses. It is possible that an exuberant innate immune response, in the form of robust inflammation, while being beneficial for resisting and surviving infectious diseases, may play a negative role in malignant transformation and cancer risk in later life [32–34]. Differences in genotype frequencies and LD structures in genes involved in mounting an inflammatory innate immune response between AA and EA women may partly explain differential risk profiles for breast cancer between these two groups. Moreover, the same genetic variants may have different effects in the two populations due to interactions with host and environmental factors that are differentially distributed between the two races [35], although this was not a focus of our study. A number of differences by menopausal status were also observed in our study consistent with the possibility that immune-related etiologic pathways for pre- and postmenopausal breast cancer may be different in many respects [36], perhaps due to the role of sex hormones in modulating both the innate and subsequent adaptive immune response [37]. Overlaid on this are potential racial differences in exposure to sex hormones that can further modulate relationships with breast cancer risk and phenotype, with AA women having higher lifetime fertility rates [38], and higher circulating estrogens compared to EA women [39], potentially due in part to higher rates of obesity in this group [40].

The strongest association observed in the study was for *TNFA*- rs1799724 in EA women, with combined CT+TT genotypes associated with a 2.3-fold increased breast cancer risk in premenopausal women, and a 2-fold increased risk of ER positive cancers. This variant located within the promoter region of the gene has been associated with increased [41] and decreased *TNFα* [42] production, as well as increased risk of radiation-induced toxicity after treatment for lung cancer [43]. Associations with breast cancer risk have not been examined in either AA or EA women, although two studies found no association within Asian populations [44,45]. *TNFα* is implicated in chronic inflammation and can support tumor growth and breast cancer progression. Positive cross-talk between 17β-estradiol and *TNFα* in inflammatory and angiogenic pathways, and the ability of *TNFα* to modulate gene regulation by 17β-estradiol may explain, in part, why associations were strongest for premenopausal EA women and ER positive breast cancers [46]. Because this variant was much less common in AA women, with an allele frequency of 7% in AAs compared to 35.6% in EAs, examination of associations among AA women was hampered by sample size. Similarly, limited sample size prevented adequate examination of potential relationships between this variant and risk of ER negative breast cancer. We also considered 4 other SNPs (rs361525, rs1800629, rs1799964, rs1800630) located in the promoter region of *TNFA*, but no significant associations were found in EA women, similar to findings from other studies [18,20,47]. *TNFA*-rs361525 was associated with a modest increase in breast cancer risk among EAs in a study of ~5300 cases and 4900 controls [21], but this was not replicated within the Breast Cancer Association Consortium of 30,000 breast cancer cases and 30,000 controls [20]. A recent Meta-Analysis reported a small decreased risk of breast cancer (OR=0.91, 95% CI, 0.85-0.97) associated with the *TNFA*- rs1800629 variant in Caucasians, although this appeared to be largely driven by results from one study (OR=0.55); no associations were observed with *TNFA*-rs1799964, rs1800630, or rs361525 [48]. In our study, *TNFA*-rs1799964 and rs1800630 were associated with a similar 1.7-fold increased breast cancer risk in premenopausal AA women who carry the variant C allele of *TNFA*-rs1799964 or the variant A allele of *TNFA*-rs1800630, but not among EA women.

*CRP* is an inflammatory effector that has been linked to breast cancer risk and poorer prognosis, with higher circulating levels observed in AA women compared to EAs [49]. Among premenopausal women, the CT and TT genotypes of *CRP*-rs1205 were associated with decreased breast cancer risk in EA, but not AA women. Located in the 3’ untranslated region of the gene, this variant is consistently associated with decreased serum *CRP* levels [50,51], but was not associated with breast cancer risk in a prospective cohort of ~3,800 EA women [52]. The total number of breast cancers diagnosed in that study, however, was limited to 172 events, and all women were 55 years of age and therefore might not have shown associations with this genetic variant if the effect is confined to younger women, as suggested by our findings. *CXCL5*, which encodes for epithelial neutrophil-activating peptide (ENA-78), is up-regulated in breast tumors and plays a role in regulating neutrophil homeostasis, an essential component of innate immunity, and a major contributor to inflammation-associated tissue damage [53]. In our study, the *CXCL5*-rs425535 variant A allele was associated with a decreased risk of breast cancer in AA premenopausal women, but not EAs, and was much more common among AA (MAF= 39.7%) than among EA women (MAF=13.5%). This SNP is located in an exon splicing enhancer site that could be important for transcriptional control and is highly linked with the *CXCL5*-rs352046 variant (r^2^=0.94), which is associated with significantly higher ENA-78 plasma concentrations [54]. Why this might translate to lower breast cancer risk among AAs is unclear and warrants further study, especially given understanding that AAs have reduced absolute neutrophil counts compared with EAs [55].

*CCL2* is an important inflammatory chemokine involved in macrophage recruitment and expression of angiogenic factors that are highly expressed within breast tumors and associated with the development and progression of breast cancer [56]. In our study, several *CCL2* SNPs (rs1024611, rs13900, rs4586) in LD were suggested to be associated with increased risk of premenopausal breast cancer in AA women, although an elevated risk observed for rs4586 in EA women in the initial analysis was attenuated towards the null in our second stage analysis using a larger study population. Of these, rs1024611 located in the promoter region of the gene has been demonstrated to affect *CCL2* protein levels by changing transcription factor binding sites, with the variant C allele being associated with higher *CCL2* levels [57]. The other two SNPs may also similarly alter *CCL2* levels. Studies have shown that *CCL2* gene expression is inhibited by 17β-estradiol [58,59], our findings may indicate that this genetic polymorphism, associated with higher levels of *CCL2*, is most relevant with respect to breast cancer risk against a background of low *CCL2* expression. Others, like us, did not observe associations between *CCL2*-rs1024611 and breast cancer risk among Caucasians [60,61].

Among postmenopausal women, two SNPs in high LD in the *IL1B* gene, rs1143627 and rs16944, were associated with an increased risk of breast cancer in EA, but not AA women. Pro-inflammatory IL-1 is implicated in cancer progression, and intratumoral levels of *IL1β* are higher compared with normal adjacent breast tissue [62]. These SNPs are located in the promoter region of the gene and the *IL1*-rs16944 variant CT/TT genotypes are associated with higher *CRP* levels than the CC genotype in healthy individuals [63]. The *IL1B*-rs1143627 variant has been associated with increased breast cancer risk in Asian populations [64,65], but has not been examined in either an EA or AA population. Several hospital-based case-control studies have examined the SNP rs16944 in Caucasians but no associations were found [66,67]. Because *IL-1β* levels in breast tissue might be controlled in vivo by estradiol and is correlated with abdominal subcutaneous fat [62], genetic polymorphisms in this gene might be most relevant among postmenopausal women, as suggested by our findings, since peripheral fat is a main source of estrogen production after menopause. The *NOD2*-rs2066842 variant was associated with decreased breast cancer risk among postmenopausal EA women, and reduced overall risk of ER negative cancers. Although this SNP has not been implicated in risk of breast cancer, it has been shown to be associated with Crohn’s disease in Caucasians [68].

Several other SNPs in *CCL2*, *CCL5*, *NOD2, FGF2* were found to be associated with either risk of ER positive or negative breast cancer in either AA or EA women, although only the *NOD2* SNP association with ER positive tumors differed between AA and EA women, and none have been examined in relation to breast cancer risk. Notably, variant alleles for two SNPs (rs2107538, rs3817655) in high LD in *CCL5* (*RANTES*) were associated with approximately 35% decreased risk of ER negative breast cancer in AA women. *RANTES* is a chemokine that assists in the recruitment of inflammatory cells, and evolutionary pressures have been shown to have significant impact on genetic variation in this gene across various populations [69]. Tumor expression of *CCL5* promotes breast cancer progression [70], and both SNPs have been associated with decreased risk of prostate cancer in men of African descent [71]. Overall, we did not identify SNPs associated with strong increased risk with ER negative tumors in AA women in our study, and this could be due to our limited statistical power in these analyses. Another potential reason could be due to our SNP selection strategy, based upon existing literature, which focused primarily on Caucasians.

One limitation of this study is that we focused on a select panel of SNPs in each gene thought to be important in cancer risk based on previous studies without including a comprehensive set of variants. This candidate gene and SNP selection approach based on limited literature for AA populations could have affected our ability to identify novel genetic variants, especially for AA women. In addition, although this is a study with a large number of AA and EA women to examine racial differences for these genetic variants with breast cancer risk, our sample size was limited when analyses were stratified by menopausal and ER status. We were able, however, to test and strengthen several promising relationships in a larger study sample after additional participant accrual. In the larger sample, controls were slightly younger than cases (stage II dataset) because eligibility criteria for cases and controls, originally up to age 65, was expanded to include older women up to age 75, fewer older controls were recruited towards the end of the study. This difference, however, was unlikely to confound or bias our findings given that all models included age as a covariate, and we performed a sensitivity analysis excluding older women (70 years old) and found results were very similar to those reported using all participants. Finally, it must be emphasized that our findings should be interpreted with caution because few associations remained significant after correction for multiple testing. Nevertheless, we expect the probability of making a type I error is attenuated by our choice to evaluate primarily functional SNPs shown to affect gene expression by changing transcriptional binding sites.

In summary, this is the most comprehensive study that was designed to specifically examine putatively functional genetic variants in the innate immunity related inflammatory pathway with breast cancer risk and risk of ER-positive and ER-negative disease in AA and EA women simultaneously. Our findings indicate that genetic variants in innate-immunity pathways are associated with breast cancer risk in both AA and EA women, although susceptible and protective loci differed by race, and played a role in the etiology of both ER-negative as well as ER-positive breast cancers. Nevertheless, as the first study to comprehensively assess these genetic variants in both AA and EA women by menopausal or ER status, our findings could provide valuable information for better understanding of the etiology of this disease in both AA and EA women. Future studies with comprehensive resequencing or functional analyses are needed to further explore these associations.

## Supporting Information

Table S1 **Characteristics of 1,307 European American (EA) and 1,365 African American (AA) cases and controls in the Women’s Health Circle of Study (WCHS).**
(PDF)

Table S2 **Single nucleotide polymorphisms (SNPs) of innate immune response related pathways and risk of breast cancer among 650 European American and 864 African American women in the WCHS.**
(PDF)

Table S3 **Single nucleotide polymorphisms (SNPs) of innate immune response related pathways and risk of breast cancer by menopausal status among 650 European American and 864 African American women in the WCHS.**
(PDF)

Table S4 **Single nucleotide polymorphisms (SNPs) of innate immune response related pathways and risk of breast cancer by estrogen receptor status among 650 European American and 864 African American women in the WCHS.**
(PDF)
