# Supplementary material for: Innate Immunity Pathways and Breast Cancer Risk in African American and European-American Women in the Women’s Circle of Health Study (WCHS)
Source: PLoS One. 2013 Aug 21;8(8):e72619. doi: 10.1371/journal.pone.0072619 (PMC3749137; doi:10.1371/journal.pone.0072619)
Supplement: Table S1 — Characteristics of 1,307 European American (EA) and 1,365 African American (AA) cases and controls in the Women’s Health Circle of Study (WCHS). (PDF) [file pone.0072619.s001.pdf]

Table S1. Characteristics of 1,307 European American (EA) and 1,365 African American (AA) cases and controls in the Women's Health Circle of Study (WCHS)[a]

Characteristics	European American			African American		
	Cases (n=658)	Controls (n=649)	*P-value*[c]	Cases (n=621)	Controls (n=744)	*P-value*[c]
Age (yr), mean (SD)[b]	52 (10.0)	49.7 (8.7)	<0.0001	51.4 (10.4)	48.6 (9.4)	<0.0001
Body mass index, mean (SD)[b]	27.2 (6.6)	27.4 (7.2)	0.58	31.1 (6.7)	31.9 (7.8)	0.03
Number of full-term pregnancy	1.5	1.6	0.77	2.2	2.2	0.76
Menopausal status, n (%)			0.32			0.04
Premenopausal	343 (52.1)	356 (54.8)		309 (49.8)	412 (55.4)	
Postmenopausal	315 (47.9)	293 (45.2)		312 (50.2)	332 (44.6)	
Family history, n (%)			0.0006			0.15
No	497 (75.5)	540 (83.2)		532 (85.7)	657 (88.3)	
Yes	161 (24.5)	109 (16.8)		89 (14.3)	87 (11.7)	
Education, n (%)			<0.0001			0.16
Less than high school	20 (3.0)	6 (0.9)		84 (13.5)	103 (13.8)	
High school	115 (17.5)	67 (10.3)		196 (31.6)	192 (25.8)	
Some college	144 (21.9)	117 (18.0)		167 (26.9)	205 (27.6)	
College graduate	201 (30.6)	210 (32.4)		107 (17.2)	149 (20.0)	
Post-graduate degree	178 (27.0)	249 (38.4)		67 (10.8)	95 (12.8)	
History of Benign Breast Disease, n (%)			0.0009			<0.0001
No	382 (58.7)	437 (67.5)		426 (68.8)	586 (78.9)	
Yes	269 (41.3)	210 (32.5)		193 (31.2)	157 (21.1)	
Breast Feeding, n (%)			0.01			0.74
Nulliparous	207 (31.5)	185 (28.5)		101 (16.3)	112 (15.0)	
No	169 (25.7)	134 (20.7)		260 (41.9)	325 (43.7)	
Yes	282 (42.9)	330 (50.8)		260 (41.9)	307 (41.3)	
Smoking Status, n (%)			0.29			<0.0001
Never Smoker	340 (51.8)	361 (55.6)		389 (62.6)	425 (57.1)	
Former Smoker	239 (36.4)	210 (32.4)		147 (23.7)	141 (19.0)	
Current Smoker	78 (11.9)	78 (12.0)		85 (13.7)	178 (23.9)	
Estrogen receptor (ER) Status [c]						<0.0001
Positive	386 (58.7)			323 (52.0)		
Negative	82 (12.4)			150 (24.2)		
Unknown/missing	190 (28.9)			148 (23.8)		

[a] Number may not add up to the total number due to missing values

[b] SD: standard deviation.

[c] *P*-value were from t-test for continuous variables and Chi-square test for categorical variables
