# Supplementary material for: Innate Immunity Pathways and Breast Cancer Risk in African American and European-American Women in the Women’s Circle of Health Study (WCHS)
Source: PLoS One. 2013 Aug 21;8(8):e72619. doi: 10.1371/journal.pone.0072619 (PMC3749137; doi:10.1371/journal.pone.0072619)
Supplement: Table S2 — Single nucleotide polymorphisms (SNPs) of innate immune response related pathways and risk of breast cancer among 650 European American and 864 African American women in the WCHS. (PDF) [file pone.0072619.s002.pdf]

Table S2. Single nucleotide polymorphisms (SNPs) of innate immune response related pathways and risk of breast cancer among 650 European American and 864 African American women in the WCHS

Gene	SNP	Chr	Coordinate	Genotype	European American			African American		
					# Case vs Control	OR (95% CI)[a,b]	$P^{c,d}$	# Case vs Control	OR (95% CI)[a,b]	$P^{c,d}$
TNFA	rs1799964	6	31542308	TT	198/198	1.00 (ref)	0.43	310/286	1.00 (ref)	0.56
				TC	111/96	1.18 (0.83-1.68)		123/102	1.13 (0.82-1.55)	
				CC	15/13	1.11 (0.49-2.49)		21/16	1.03 (0.51-2.05)	
				TC/CC	126/109	1.17 (0.83-1.64)	0.37	144/118	1.11 (0.83-1.50)	0.48
TNFA	rs1800630	6	31542476	CC	233/224	1.00 (ref)	0.61	347/320	1.00 (ref)	0.41
				CA	85/81	1.04 (0.72-1.50)		98/76	1.24 (0.88-1.75)	
				AA	10/6	1.47 (0.49-4.40)		9/8	0.86 (0.32-2.31)	
				CA/AA	95/87	1.07 (0.74-1.53)	0.72	107/84	1.20 (0.86-1.67)	0.29
TNFA	rs1799724	6	31542482	CC	215/231	1.00 (ref)	0.007	428/373	1.00 (ref)	0.59
				CT	93/66	1.69 (1.15-2.48)		26/30	0.86 (0.49-1.50)	
				TT	10/7	1.80 (0.65-5.00)		0/0	n/a	
				CT/TT	103/73	1.70 (1.17-2.46)	0.005	n/a	n/a	
TNFA	rs1800629	6	31543031	GG	233/225	1.00 (ref)	0.80	351/310	1.00 (ref)	0.56
				GA	76/80	0.94 (0.64-1.37)		96/86	0.97 (0.69-1.36)	
				AA	7/3	2.28 (0.56-9.26)		7/9	0.64 (0.23-1.80)	
				GA/AA	83/83	0.99 (0.68-1.43)	0.95	103/95	0.94 (0.67-1.30)	0.69
TNFA	rs361525	6	31543101	GG	283/276	1.00 (ref)	0.78	419/373	1.00 (ref)	0.90
				GA	33/27	1.09 (0.61-1.95)		35/29	1.03 (0.61-1.75)	
				AA	1/1	0.98 (0.06-16.43)		0/1	n/a	
				GA/AA	34/28	1.09 (0.62-1.92)	0.77	35/30	1.00 (0.59-1.69)	1.00
TNFRSF1A	rs767455	12	6450945	TT	102/96	1.00 (ref)	0.77	169/177	1.00 (ref)	0.21
				TC	162/161	0.93 (0.64-1.35)		231/176	1.38 (1.03-1.86)	
				CC	65/55	1.11 (0.69-1.80)		53/52	1.11 (0.71-1.74)	
				TC/CC	227/216	0.97 (0.68-1.39)	0.88	284/228	1.32 (0.99-1.75)	0.06
TNFRSF1A	rs4149570	12	6451590	GG	121/115	1.00 (ref)	0.40	354/314	1.00 (ref)	0.97
				GT	158/154	1.01 (0.71-1.45)		92/86	0.93 (0.65-1.32)	
				TT	50/42	1.30 (0.78-2.16)		9/5	1.62 (0.52-5.05)	
				GT/TT	208/196	1.07 (0.76-1.51)	0.69	101/91	0.96 (0.68-1.35)	0.82
CCL2	rs1024611	17	32579788	TT	164/171	1.00 (ref)	0.59	286/268	1.00 (ref)	0.65
				TC	144/116	1.26 (0.90-1.78)		160/123	1.21 (0.90-1.63)	
				CC	18/22	0.83 (0.41-1.67)		9/14	0.61 (0.25-1.45)	
				TC/CC	162/138	1.2 (0.86-1.66)	0.28	169/137	1.15 (0.86-1.54)	0.34

CCL2	rs2857656	17	32582007	GG	157/166	1.00 (ref)	0.56	161/154	1.00 (ref)	0.47
				GC	137/112	1.26 (0.89-1.79)		204/187	1.01 (0.74-1.37)	
				CC	17/21	0.85 (0.41-1.75)		76/60	1.20 (0.79-1.81)	
				GC/CC	154/133	1.20 (0.86-1.68)	0.28	280/247	1.05 (0.79-1.41)	0.72
CCL2	rs4586	17	32583269	TT	109/141	1.00 (ref)	0.12	52/45	1.00 (ref)	0.13
				TC	168/128	1.67 (1.16-2.4)		189/203	0.81 (0.51-1.28)	
				CC	35/37	1.12 (0.63-1.97)		207/156	1.16 (0.72-1.85)	
				TC/CC	203/165	1.54 (1.10-2.18)	0.01	396/359	0.95 (0.61-1.48)	0.83
CCL2	rs13900	17	32583911	CC	164/171	1.00 (ref)	0.49	286/266	1.00 (ref)	0.78
				CT	144/117	1.26 (0.89-1.77)		160/123	1.21 (0.90-1.62)	
				TT	18/20	0.90 (0.44-1.85)		7/14	0.49 (0.19-1.25)	
				CT/TT	162/137	1.21 (0.87-1.68)	0.26	167/137	1.14 (0.85-1.52)	0.39
CCL5	rs3817655	17	34199641	TT	209/202	1.00 (ref)	0.91	155/131	1.00 (ref)	0.79
				TA	99/98	0.97 (0.68-1.40)		205/198	0.83 (0.61-1.14)	
				AA	9/8	1.01 (0.36-2.81)		95/76	0.99 (0.67-1.47)	
				TA/AA	108/106	0.98 (0.69-1.39)	0.89	300/274	0.88 (0.65-1.17)	0.38
CCL5	rs2280789	17	34207003	TT	234/236	1.00 (ref)	0.41	291/267	1.00 (ref)	0.38
				TC	91/72	1.25 (0.86-1.84)		142/114	1.16 (0.85-1.58)	
				CC	4/5	0.73 (0.18-2.99)		20/17	1.12 (0.56-2.22)	
				TC/CC	95/77	1.22 (0.84-1.77)	0.30	162/131	1.16 (0.86-1.55)	0.33
CCL5	rs2107538	17	34207780	CC	205/200	1.00 (ref)	0.81	156/131	1.00 (ref)	0.83
				CT	103/94	1.11 (0.77-1.59)		205/199	0.83 (0.60-1.13)	
				TT	8/9	0.82 (0.29-2.28)		93/73	1.01 (0.68-1.51)	
				CT/TT	111/103	1.08 (0.76-1.53)	0.67	298/272	0.88 (0.65-1.17)	0.37
CXCL5	rs425535	4	74863997	GG	245/240	1.00 (ref)	0.88	168/142	1.00 (ref)	0.30
				GA	65/63	0.97 (0.64-1.46)		224/198	0.93 (0.68-1.26)	
				AA	8/5	1.39 (0.42-4.60)		63/65	0.80 (0.52-1.22)	
				GA/AA	73/68	1.00 (0.67-1.48)	0.99	287/263	0.89 (0.67-1.19)	0.45
CRP	rs1205	1	159682233	CC	145/118	1.00 (ref)	0.22	281/257	1.00 (ref)	0.81
				CT	137/149	0.77 (0.54-1.1)		155/121	1.20 (0.88-1.62)	
				TT	33/35	0.80 (0.46-1.41)		18/25	0.58 (0.30-1.11)	
				CT/TT	170/184	0.78 (0.55-1.09)	0.15	173/146	1.09 (0.81-1.45)	0.58
CRP	rs1130864	1	159683091	CC	150/157	1.00 (ref)	0.25	290/266	1.00 (ref)	0.64
				CT	134/118	1.19 (0.84-1.69)		99/89	0.99 (0.71-1.4)	
				TT	32/24	1.31 (0.71-2.40)		15/18	0.78 (0.38-1.59)	
				CT/TT	166/142	1.21 (0.87-1.69)	0.26	114/107	0.96 (0.69-1.32)	0.79

CRP	rs1800947	1	159683438	GG	280/262	1.00 (ref)	0.63	450/392	1.00 (ref)	0.04
				GC	37/40	0.93 (0.56-1.53)		4/11	0.29 (0.09-0.96)	
				CC	1/2	0.49 (0.04-5.99)		0/0	n/a	
				GC/CC	38/42	0.91 (0.55-1.49)	0.70	n/a	n/a	
IL1A	rs17561	2	113537223	GG	158/154	1.00 (ref)	0.94	311/287	1.00 (ref)	0.44
				GT	138/118	1.30 (0.92-1.85)		131/108	1.15 (0.84-1.57)	
				TT	32/41	0.81 (0.47-1.38)		12/10	1.08 (0.45-2.58)	
				GT/TT	170/159	1.17 (0.84-1.63)	0.35	143/118	1.14 (0.84-1.55)	0.40
IL1A	rs1800587	2	113542960	CC	155/153	1.00 (ref)	0.82	163/155	1.00 (ref)	0.62
				CT	138/116	1.3 (0.91-1.84)		215/186	1.08 (0.80-1.46)	
				TT	32/39	0.85 (0.49-1.46)		76/64	1.09 (0.72-1.65)	
				CT/TT	170/155	1.18 (0.85-1.64)	0.32	291/250	1.08 (0.81-1.44)	0.59
IL1B	rs1143634	2	113590390	CC	181/180	1.00 (ref)	0.80	333/306	1.00 (ref)	0.13
				CT	125/103	1.32 (0.93-1.88)		108/96	1.06 (0.76-1.47)	
				TT	20/26	0.72 (0.38-1.37)		14/3	4.42 (1.23-15.86)	
				CT/TT	145/129	1.19 (0.86-1.66)	0.30	122/99	1.16 (0.84-1.59)	0.37
IL1B	rs1143627	2	113594387	TT	137/145	1.00 (ref)	0.19	72/74	1.00 (ref)	0.75
				TC	141/134	1.14 (0.81-1.62)		218/178	1.30 (0.88-1.93)	
				CC	50/33	1.41 (0.83-2.39)		165/153	1.14 (0.76-1.72)	
				TC/CC	191/167	1.20 (0.86-1.66)	0.28	383/331	1.23 (0.85-1.78)	0.27
IL1B	rs16944	2	113594867	GG	137/143	1.00 (ref)	0.20	90/93	1.00 (ref)	0.72
				GA	140/134	1.11 (0.79-1.58)		229/185	1.30 (0.91-1.87)	
				AA	49/32	1.44 (0.84-2.46)		131/125	1.11 (0.75-1.64)	
				GA/AA	189/166	1.18 (0.85-1.64)	0.33	360/310	1.23 (0.88-1.72)	0.24
IL1R1	rs2041748	2	102756295	CC	193/178	1.00 (ref)	0.57	389/349	1.00 (ref)	0.75
				CT	122/118	0.99 (0.70-1.39)		60/52	1.12 (0.74-1.71)	
				TT	14/17	0.71 (0.33-1.55)		4/4	0.77 (0.18-3.32)	
				CT/TT	136/135	0.95 (0.68-1.32)	0.76	64/56	1.10 (0.73-1.65)	0.65
IL1R2	rs2228139	2	102781649	CC	297/281	1.00 (ref)	0.96	405/365	1.00 (ref)	0.92
				CG	28/27	0.93 (0.52-1.67)		47/39	1.06 (0.67-1.67)	
				GG	1/0			1/1	0.50 (0.03-8.64)	
				CG/GG	29/27	0.97 (0.55-1.73)	0.92	48/40	1.04 (0.66-1.64)	0.86
IL1RN	rs4251961	2	113874467	TT	135/115	1.00 (ref)	0.36	310/266	1.00 (ref)	0.24
				TC	138/147	0.8 (0.56-1.16)		130/123	0.88 (0.65-1.2)	
				CC	44/46	0.86 (0.51-1.43)		13/16	0.66 (0.3-1.45)	
				TC/CC	182/193	0.82 (0.58-1.15)	0.24	143/139	0.86 (0.64-1.16)	0.32

IL1RN	rs419598	2	113887207	TT	167/166	1.00 (ref)	0.81	354/305	1.00 (ref)	0.49
				TC	119/106	1.23 (0.86-1.76)		73/73	0.88 (0.60-1.27)	
				CC	24/29	0.85 (0.46-1.56)		6/6	0.88 (0.27-2.89)	
				TC/CC	143/135	1.14 (0.82-1.60)	0.43	79/79	0.88 (0.61-1.25)	0.47
IL1RN	rs454078	2	113888793	AA	170/164	1.00 (ref)	0.71	395/344	1.00 (ref)	0.37
				AT	133/123	1.11 (0.79-1.57)		57/57	0.85 (0.57-1.29)	
				TT	26/25	1.01 (0.55-1.88)		3/4	0.65 (0.14-3.12)	
				AT/TT	159/148	1.09 (0.79-1.52)	0.59	60/61	0.84 (0.56-1.26)	0.40
IL1RN	rs579543	2	113889631	CC	173/159	1.00 (ref)	0.54	355/320	1.00 (ref)	0.79
				CT	132/127	0.97 (0.69-1.37)		94/80	1.08 (0.76-1.53)	
				TT	23/26	0.78 (0.41-1.46)		5/5	0.84 (0.24-3.00)	
				CT/TT	155/153	0.94 (0.68-1.30)	0.71	99/85	1.07 (0.76-1.50)	0.71
IL6	rs10499563	7	22760488	TT	204/201	1.00 (ref)	0.77	289/261	1.00 (ref)	0.54
				TC	114/96	1.13 (0.79-1.6)		143/125	1.07 (0.79-1.45)	
				CC	11/14	0.85 (0.36-2.00)		22/16	1.19 (0.60-2.35)	
				TC/CC	125/110	1.09 (0.78-1.54)	0.60	165/141	1.08 (0.81-1.45)	0.58
IL6	rs1800797	7	22766221	GG	151/123	1.00 (ref)	0.43	401/352	1.00 (ref)	0.73
				GA	143/158	0.78 (0.55-1.1)		48/47	0.86 (0.55-1.36)	
				AA	35/31	0.96 (0.55-1.70)		2/0		
				GA/AA	178/189	0.81 (0.58-1.13)	0.21	50/47	0.89 (0.57-1.40)	0.61
IL6	rs1800796	7	22766246	GG	281/269	1.00 (ref)	0.75	348/319	1.00 (ref)	0.32
				GC	48/41	1.09 (0.68-1.76)		95/82	1.05 (0.75-1.48)	
				CC	0/3			11/4	2.40 (0.74-7.83)	
				GC/CC	48/44	1.01 (0.63-1.61)	0.97	106/86	1.11 (0.80-1.55)	0.52
IL6	rs1800795	7	22766645	GG	148/123	1.00 (ref)	0.67	405/356	1.00 (ref)	0.48
				GC	143/152	0.84 (0.59-1.19)		47/48	0.82 (0.52-1.29)	
				CC	37/33	1.01 (0.58-1.75)		2/1	1.43 (0.12-17.13)	
				GC/CC	180/185	0.87 (0.62-1.21)	0.41	49/49	0.83 (0.53-1.30)	0.43
IL6R	rs2228145	1	154426970	AA	124/117	1.00 (ref)	0.59	345/292	1.00 (ref)	0.22
				AC	153/145	0.98 (0.69-1.40)		102/104	0.83 (0.60-1.16)	
				CC	52/48	1.20 (0.73-1.96)		7/9	0.71 (0.25-2.01)	
				AC/CC	205/193	1.03 (0.74-1.44)	0.87	109/113	0.82 (0.6-1.14)	0.24
IL8	rs4073	4	74606024	TT	113/106	1.00 (ref)	0.87	13/22	1.00 (ref)	0.56
				TA	151/150	0.91 (0.63-1.32)		160/138	1.96 (0.93-4.12)	
				AA	53/52	0.99 (0.61-1.61)		273/244	1.83 (0.87-3.83)	
				TA/AA	204/202	0.93 (0.66-1.32)	0.69	433/382	1.89 (0.91-3.9)	0.09

IL8	rs2227306	4	74607055	CC	142/127	1.00 (ref)	0.87	350/312	1.00 (ref)	0.89
				CT	128/128	0.96 (0.66-1.38)		78/63	1.11 (0.76-1.62)	
				TT	40/42	0.98 (0.58-1.65)		5/7	0.70 (0.21-2.31)	
				CT/TT	168/170	0.96 (0.68-1.35)	0.82	83/70	1.07 (0.74-1.54)	0.73
IL16	rs4778889	15	81588995	TT	225/203	1.00 (ref)	0.26	153/146	1.00 (ref)	0.50
				TC	91/92	0.85 (0.59-1.22)		225/192	1.13 (0.83-1.53)	
				CC	11/14	0.68 (0.27-1.71)		69/65	1.12 (0.74-1.7)	
				TC/CC	102/106	0.83 (0.58-1.18)	0.29	294/257	1.13 (0.84-1.5)	0.43
IL17A	rs7747909	6	52054249	GG	186/175	1.00 (ref)	0.94	375/326	1.00 (ref)	0.52
				GA	125/112	1.15 (0.81-1.62)		73/74	0.88 (0.61-1.27)	
				AA	15/22	0.77 (0.38-1.58)		6/5	0.94 (0.27-3.29)	
				GA/AA	140/134	1.09 (0.78-1.51)	0.62	79/79	0.88 (0.62-1.26)	0.49
IL17A	rs1974226	6	52055335	GG	216/201	1.00 (ref)	0.94	355/320	1.00 (ref)	0.47
				GA	97/101	0.92 (0.64-1.31)		87/73	1.12 (0.79-1.6)	
				AA	15/11	1.21 (0.53-2.78)		11/6	1.22 (0.43-3.41)	
				GA/AA	112/112	0.95 (0.67-1.33)	0.76	98/79	1.13 (0.8-1.59)	0.48
FGF2	rs308379	4	123782896	AA	136/145	1.00 (ref)	0.10	300/289	1.00 (ref)	0.24
				AT	142/125	1.3 (0.91-1.85)		136/102	1.22 (0.89-1.68)	
				TT	47/39	1.43 (0.86-2.38)		15/12	1.17 (0.53-2.61)	
				AT/TT	189/164	1.33 (0.95-1.85)	0.09	151/114	1.22 (0.9-1.65)	0.20
FGF2	rs1048201	4	123814308	CC	221/217	1.00 (ref)	0.17	395/362	1.00 (ref)	0.45
				CT	86/79	1.15 (0.79-1.67)		57/40	1.25 (0.8-1.94)	
				TT	19/13	1.69 (0.8-3.59)		2/2	0.7 (0.1-5.14)	
				CT/TT	105/92	1.22 (0.86-1.74)	0.26	59/42	1.22 (0.79-1.88)	0.38
NOD2	rs2066842	16	50744624	CC	184/168	1.00 (ref)	0.09	400/364	1.00 (ref)	0.22
				CT	119/121	0.77 (0.54-1.1)		54/38	1.49 (0.94-2.39)	
				TT	13/19	0.62 (0.29-1.34)		1/3	0.37 (0.04-3.98)	
				CT/TT	132/140	0.75 (0.54-1.06)	0.10	55/41	1.43 (0.9-2.27)	0.13

[a] OR, odds ratio; 95%CI, 95% confidence interval

[b] Adjusted for age at diagnosis, education, body mass index, family history of breast cancer, history of benign breast disease, smoking status, menopausal status, and proportion of European ancestry.

[c] *P*-trend for genetic dose response determined by coding genotypes as having 0, 1, or 2 variant allele, which was subsequently analyzed as an ordinal variable.

[d] *P* for heterogeneity from dominant models (heterozygous and homozygous variant combined vs. homozygous common).
