# Supplementary material for: Innate Immunity Pathways and Breast Cancer Risk in African American and European-American Women in the Women’s Circle of Health Study (WCHS)
Source: PLoS One. 2013 Aug 21;8(8):e72619. doi: 10.1371/journal.pone.0072619 (PMC3749137; doi:10.1371/journal.pone.0072619)
Supplement: Table S3 — Single nucleotide polymorphisms (SNPs) of innate immune response related pathways and risk of breast cancer by menopausal status among 650 European American and 864 African American women in the WCHS. (PDF) [file pone.0072619.s003.pdf]

Table S3. Single nucleotide polymorphisms (SNPs) of innate immune response related pathways and risk of breast cancer by menopausal status among 650 European American and 864 African American women in the WCHS

Gene	SNP	Genotype	European American						African American					
			Pre-menopausal women			Post-menopausal women			Pre-menopausal women			Post-menopausal women		
			# Case/ Control	OR (95% CI)[a,b]	*P*[c,d]	# Case / Control	OR (95% CI) [a,b]	*P*[c,d]	# Case / Control	OR (95% CI) [a,b]	*P*[c,d]	# Case / Control	OR (95% CI) [a,b]	*P*[c,d]
TNFA	rs1799964	TT	112/102	1.00 (ref)	0.58	86/96	1.00 (ref)	0.43	155/145	1.00 (ref)	0.09	155/141	1.00 (ref)	0.45
		TC	64/55	1.19 (0.72-1.94)		47/41	1.24 (0.73-2.10)		73/40	1.86 (1.17-2.95)		50/62	0.75 (0.47-1.19)	
		CC	8/6	1.06 (0.32-3.46)		7/7	1.26 (0.39-4.03)		11/10	0.95 (0.37-2.40)		10/6	1.14 (0.38-3.39)	
		TC/CC	72/61	1.17 (0.73-1.89)	0.51	54/48	1.24 (0.75-2.06)	0.40	84/50	1.67 (1.08-2.57)	0.02	60/68	0.79 (0.51-1.22)	0.29
TNFA	rs1800630	CC	133/115	1.00 (ref)	0.75	100/109	1.00 (ref)	0.26	179/159	1.00 (ref)	0.12	168/161	1.00 (ref)	0.75
		CA	46/46	0.91 (0.54-1.52)		39/35	1.24 (0.71-2.15)		54/29	1.86 (1.11-3.14)		44/47	0.91 (0.55-1.48)	
		AA	6/4	0.97 (0.23-4.11)		4/2	2.55 (0.42-15.41)		5/6	0.77 (0.22-2.69)		4/2	1.05 (0.17-6.34)	
		CA/AA	52/50	0.91 (0.55-1.50)	0.72	43/37	1.30 (0.76-2.23)	0.34	59/35	1.67 (1.02-2.72)	0.04	48/49	0.91 (0.56-1.48)	0.71
TNFA	rs1799724	CC	118/128	1.00 (ref)	0.004	97/103	1.00 (ref)	0.73	224/184	1.00 (ref)	0.24	204/189	1.00 (ref)	0.08
		CT	56/28	2.30 (1.31-4.04)		37/38	1.11 (0.63-1.95)		15/10	1.68 (0.70-4.02)		11/20	0.49 (0.22-1.09)	
		TT	7/4	2.38 (0.61-9.32)		3/3	1.07 (0.20-5.66)		0/0	n/a		0/0	n/a	
		CT/TT	63/32	2.31 (1.35-3.95)	0.002	40/41	1.11 (0.64-1.92)	0.71		n/a			n/a	
TNFA	rs1800629	GG	138/110	1.00 (ref)	0.35	95/115	1.00 (ref)	0.09	188/152	1.00 (ref)	0.60	163/158	1.00 (ref)	0.75
		GA	38/49	0.67 (0.39-1.14)		38/31	1.50 (0.85-2.65)		46/38	0.91 (0.55-1.50)		50/48	1.00 (0.62-1.61)	
		AA	4/2	1.94 (0.30-12.60)		3/1	3.36 (0.33-34.07)		5/5	0.76 (0.21-2.80)		2/4	0.54 (0.09-3.19)	
		GA/AA	42/51	0.71 (0.42-1.20)	0.20	41/32	1.56 (0.89-2.73)	0.12	51/43	0.89 (0.55-1.44)	0.64	52/52	0.96 (0.60-1.54)	0.87
TNFA	rs361525	GG	158/148	1.00 (ref)	0.15	125/128	1.00 (ref)	0.42	220/181	1.00 (ref)	0.81	199/192	1.00 (ref)	0.71
		GA	22/12	1.84 (0.80-4.25)		11/15	0.64 (0.26-1.53)		19/13	1.09 (0.51-2.33)		16/16	0.98 (0.45-2.11)	
		AA	0/0	n/a		1/1	1.22 (0.07-22.45)		0/0	n/a		0/1	n/a	
		GA/AA		n/a		12/16	0.66 (0.28-1.56)	0.35		n/a		16/17	0.92 (0.43-1.96)	0.83
TNFRSF1A	rs767455	TT	58/54	1.00 (ref)	0.56	44/42	1.00 (ref)	0.88	83/86	1.00 (ref)	0.15	86/91	1.00 (ref)	0.78
		TC	95/91	0.91 (0.55-1.52)		67/70	0.91 (0.52-1.59)		129/86	1.59 (1.05-2.42)		102/90	1.22 (0.79-1.88)	
		CC	33/21	1.37 (0.66-2.82)		32/34	0.96 (0.49-1.89)		27/23	1.23 (0.64-2.36)		26/29	0.96 (0.51-1.81)	
		TC/CC	128/112	0.99 (0.61-1.62)	0.98	99/104	0.92 (0.55-1.56)	0.76	156/109	1.51 (1.01-2.26)	0.04	128/119	1.16 (0.77-1.74)	0.48
TNFRSF1A	rs4149570	GG	58/57	1.00 (ref)	0.25	63/58	1.00 (ref)	0.91	190/155	1.00 (ref)	0.95	164/159	1.00 (ref)	0.91
		GT	101/83	1.42 (0.85-2.38)		57/71	0.74 (0.43-1.25)		46/37	1.01 (0.61-1.68)		46/49	0.82 (0.50-1.35)	
		TT	27/25	1.39 (0.68-2.85)		23/17	1.29 (0.61-2.72)		3/3	1.07 (0.20-5.85)		6/2	2.51 (0.45-13.82)	
		GT/TT	128/108	1.41 (0.86-2.32)	0.17	80/88	0.84 (0.51-1.39)	0.50	49/40	1.01 (0.61-1.67)	0.97	52/51	0.88 (0.54-1.43)	0.61

CCL2	rs1024611	TT	99/96	1.00 (ref)	0.60	65/75	1.00 (ref)	0.65	147/138	1.00 (ref)	0.10	139/130	1.00 (ref)	0.31
		TC	77/55	1.27 (0.78-2.05)		67/61	1.29 (0.78-2.12)		86/53	1.48 (0.96-2.28)		74/70	0.96 (0.63-1.46)	
		CC	9/12	0.93 (0.35-2.46)		9/10	0.84 (0.29-2.42)		6/4	1.29 (0.34-4.81)		3/10	0.33 (0.08-1.29)	
		TC/CC	86/67	1.21 (0.76-1.92)	0.42	76/71	1.23 (0.76-2.00)	0.41	92/57	1.47 (0.96-2.24)	0.07	77/80	0.88 (0.58-1.34)	0.56
CCL2	rs2857656	GG	93/94	1.00 (ref)	0.55	64/72	1.00 (ref)	0.64	84/80	1.00 (ref)	0.46	77/74	1.00 (ref)	0.93
		GC	75/53	1.34 (0.82-2.20)		62/59	1.21 (0.73-2.02)		104/85	1.10 (0.71-1.70)		100/102	0.84 (0.54-1.31)	
		CC	8/12	0.86 (0.31-2.39)		9/9	0.98 (0.33-2.89)		42/30	1.24 (0.69-2.22)		34/30	1.13 (0.61-2.10)	
		GC/CC	83/65	1.27 (0.79-2.03)	0.33	71/68	1.18 (0.72-1.95)	0.51	146/115	1.14 (0.76-1.71)	0.54	134/132	0.90 (0.59-1.38)	0.64
CCL2	rs4586	TT	63/78	1.00 (ref)	0.04	46/63	1.00 (ref)	0.77	26/31	1.00 (ref)	0.05	26/14	1.00 (ref)	0.87
		TC	90/64	1.71 (1.03-2.85)		78/64	1.70 (1.00-2.90)		99/92	1.26 (0.68-2.35)		90/111	0.35 (0.17-0.74)	
		CC	23/17	1.83 (0.83-4.03)		12/20	0.68 (0.28-1.64)		110/72	1.76 (0.93-3.33)		97/84	0.53 (0.25-1.13)	
		TC/CC	113/81	1.74 (1.07-2.81)	0.03	90/84	1.45 (0.87-2.41)	0.15	209/164	1.46 (0.81-2.64)	0.21	187/195	0.43 (0.21-0.88)	0.02
CCL2	rs13900	CC	99/96	1.00 (ref)	0.54	65/75	1.00 (ref)	0.59	147/137	1.00 (ref)	0.14	139/129	1.00 (ref)	0.28
		CT	77/55	1.27 (0.78-2.05)		67/62	1.27 (0.77-2.10)		86/54	1.45 (0.94-2.24)		74/69	0.97 (0.63-1.48)	
		TT	9/11	0.99 (0.37-2.66)		9/9	0.92 (0.31-2.70)		5/4	1.09 (0.28-4.28)		2/10	0.25 (0.05-1.19)	
		CT/TT	86/66	1.22 (0.77-1.95)	0.39	76/71	1.23 (0.76-2.00)	0.41	91/58	1.43 (0.94-2.18)	0.10	76/79	0.89 (0.58-1.34)	0.57
CCL5	rs3817655	TT	121/108	1.00 (ref)	0.60	88/94	1.00 (ref)	0.99	80/63	1.00 (ref)	0.94	75/68	1.00 (ref)	0.82
		TA	54/47	0.90 (0.54-1.52)		45/51	0.92 (0.54-1.57)		113/100	0.84 (0.54-1.31)		92/98	0.78 (0.49-1.23)	
		AA	5/6	0.77 (0.21-2.85)		4/2	1.67 (0.27-10.11)		46/32	1.04 (0.58-1.86)		49/44	0.98 (0.57-1.70)	
		TA/AA	59/53	0.89 (0.54-1.46)	0.64	49/53	0.96 (0.57-1.61)	0.87	159/132	0.89 (0.58-1.35)	0.58	141/142	0.84 (0.55-1.29)	0.43
CCL5	rs2280789	TT	132/132	1.00 (ref)	0.27	102/104	1.00 (ref)	0.79	148/135	1.00 (ref)	0.14	143/132	1.00 (ref)	0.97
		TC	52/31	1.58 (0.91-2.75)		39/41	0.93 (0.54-1.60)		84/53	1.42 (0.92-2.18)		58/61	0.99 (0.62-1.56)	
		CC	2/3	0.54 (0.08-3.69)		2/2	0.94 (0.11-7.86)		7/5	1.29 (0.39-4.34)		13/12	1.05 (0.45-2.47)	
		TC/CC	54/34	1.48 (0.86-2.53)	0.16	41/43	0.93 (0.54-1.59)	0.78	91/58	1.41 (0.93-2.14)	0.11	71/73	1.00 (0.65-1.53)	0.99
CCL5	rs2107538	CC	119/108	1.00 (ref)	0.85	86/92	1.00 (ref)	0.81	81/62	1.00 (ref)	0.92	75/69	1.00 (ref)	0.87
		CT	56/46	1.06 (0.64-1.77)		47/48	1.05 (0.61-1.78)		114/103	0.82 (0.53-1.27)		91/96	0.79 (0.50-1.24)	
		TT	4/6	0.62 (0.16-2.48)		4/3	1.19 (0.24-5.88)		44/29	1.06 (0.58-1.92)		49/44	1.00 (0.58-1.72)	
		CT/TT	60/52	1.01 (0.62-1.66)	0.97	51/51	1.06 (0.63-1.78)	0.84	158/132	0.87 (0.57-1.33)	0.52	140/140	0.85 (0.56-1.30)	0.46
CXCL5	rs425535	GG	132/127	1.00 (ref)	0.22	113/113	1.00 (ref)	0.22	95/59	1.00 (ref)	0.006	73/83	1.00 (ref)	0.23
		GA	41/31	1.37 (0.77-2.44)		24/32	0.69 (0.37-1.28)		116/99	0.68 (0.44-1.05)		108/99	1.28 (0.82-2.01)	
		AA	7/3	1.67 (0.37-7.41)		1/2	0.62 (0.05-7.27)		28/37	0.43 (0.23-0.80)		35/28	1.39 (0.75-2.58)	
		GA/AA	48/34	1.40 (0.81-2.43)	0.23	25/34	0.68 (0.37-1.25)	0.21	144/136	0.61 (0.40-0.93)	0.02	143/127	1.31 (0.86-2.00)	0.21
CRP	rs1205	CC	88/53	1.00 (ref)	0.004	57/65	1.00 (ref)	0.13	150/126	1.00 (ref)	0.83	131/131	1.00 (ref)	0.96

		CT	75/79	0.61 (0.37-1.01)		62/70	1.11 (0.65-1.87)		81/54	1.36 (0.88-2.11)		74/67	1.08 (0.70-1.67)	
		TT	16/26	0.34 (0.16-0.75)		17/9	2.40 (0.95-6.04)		8/14	0.43 (0.17-1.09)		10/11	0.79 (0.30-2.06)	
		CT/TT	91/105	0.54 (0.33-0.87)	0.01	79/79	1.26 (0.76-2.08)	0.36	89/68	1.16 (0.77-1.74)	0.49	84/78	1.04 (0.69-1.58)	0.85
CRP	rs1130864	CC	79/85	1.00 (ref)	0.15	71/72	1.00 (ref)	0.76	153/129	1.00 (ref)	0.47	137/137	1.00 (ref)	0.87
		CT	80/56	1.67 (1.01-2.76)		54/62	0.87 (0.52-1.45)		54/43	0.90 (0.55-1.47)		45/46	1.01 (0.61-1.67)	
		TT	20/17	1.29 (0.58-2.86)		12/7	1.66 (0.60-4.60)		6/7	0.66 (0.21-2.09)		9/11	0.88 (0.34-2.26)	
		CT/TT	100/73	1.59 (0.99-2.55)	0.06	66/69	0.95 (0.58-1.56)	0.85	60/50	0.87 (0.54-1.38)	0.55	54/57	0.99 (0.62-1.57)	0.95
CRP	rs1800947	GG	161/137	1.00 (ref)	0.33	119/125	1.00 (ref)	0.78	235/191	1.00 (ref)	0.96	215/201	1.00 (ref)	0.98
		GC	19/22	0.70 (0.34-1.44)		18/18	1.24 (0.59-2.60)		4/3	1.04 (0.22-4.99)		0/8	n/a	
		CC	1/1	0.67 (0.04-12.69)		0/1			0/0	n/a		0/0	n/a	
		GC/CC	20/23	0.70 (0.35-1.41)	0.32	18/19	1.18 (0.57-2.45)	0.66		n/a			n/a	
IL1A	rs17561	GG	87/79	1.00 (ref)	0.81	71/75	1.00 (ref)	1.00	167/143	1.00 (ref)	0.84	144/144	1.00 (ref)	0.29
		GT	78/65	1.16 (0.71-1.89)		60/53	1.37 (0.82-2.30)		67/46	1.19 (0.75-1.89)		64/62	1.18 (0.75-1.84)	
		TT	20/22	0.78 (0.37-1.64)		12/19	0.72 (0.31-1.65)		5/6	0.65 (0.19-2.22)		7/4	1.90 (0.51-7.07)	
		GT/TT	98/87	1.06 (0.67-1.67)	0.81	72/72	1.20 (0.74-1.95)	0.46	72/52	1.12 (0.72-1.74)	0.61	71/66	1.22 (0.79-1.88)	0.38
IL1A	rs1800587	CC	86/79	1.00 (ref)	0.99	69/74	1.00 (ref)	0.96	85/79	1.00 (ref)	0.93	78/76	1.00 (ref)	0.79
		CT	78/62	1.22 (0.74-1.99)		60/54	1.32 (0.78-2.21)		118/83	1.2 0(0.78-1.86)		97/103	0.92 (0.59-1.43)	
		TT	20/21	0.84 (0.40-1.77)		12/18	0.77 (0.33-1.77)		36/33	0.88 (0.49-1.59)		40/31	1.15 (0.63-2.08)	
		CT/TT	98/83	1.12 (0.71-1.77)	0.64	72/72	1.18 (0.72-1.92)	0.51	154/116	1.11 (0.74-1.68)	0.61	137/134	0.97 (0.64-1.47)	0.89
IL1B	rs1143634	CC	100/96	1.00 (ref)	0.95	81/84	1.00 (ref)	0.79	176/146	1.00 (ref)	0.43	157/160	1.00 (ref)	0.21
		CT	72/54	1.29 (0.79-2.10)		53/49	1.23 (0.74-2.06)		55/48	0.92 (0.58-1.45)		53/48	1.24 (0.77-2.00)	
		TT	13/13	0.68 (0.28-1.68)		7/13	0.58 (0.21-1.56)		8/1	7.25 (0.88-59.8)		6/2	2.53 (0.47-13.68)	
		CT/TT	85/67	1.16 (0.73-1.84)	0.52	60/62	1.09 (0.67-1.77)	0.73	63/49	1.04 (0.66-1.63)	0.86	59/50	1.29 (0.81-2.07)	0.28
IL1B	rs1143627	TT	87/74	1.00 (ref)	0.71	50/71	1.00 (ref)	0.003	41/33	1.00 (ref)	0.87	31/41	1.00 (ref)	0.67
		TC	79/71	1.06 (0.65-1.73)		62/63	1.49 (0.88-2.52)		114/86	1.11 (0.64-1.94)		104/92	1.35 (0.77-2.38)	
		CC	20/21	0.78 (0.36-1.68)		30/12	3.50 (1.55-7.94)		84/76	0.99 (0.56-1.78)		81/77	1.21 (0.67-2.18)	
		TC/CC	99/92	0.99 (0.63-1.58)	0.98	92/75	1.80 (1.09-2.95)	0.02	198/162	1.06 (0.63-1.79)	0.83	185/169	1.29 (0.76-2.19)	0.35
IL1B	rs16944	GG	88/72	1.00 (ref)	0.63	49/71	1.00 (ref)	0.002	56/44	1.00 (ref)	0.88	34/49	1.00 (ref)	0.87
		GA	78/71	0.97 (0.60-1.58)		62/63	1.54 (0.91-2.61)		109/90	1.01 (0.61-1.66)		120/95	1.65 (0.97-2.82)	
		AA	19/20	0.80 (0.37-1.73)		30/12	3.58 (1.58-8.14)		72/61	1.04 (0.60-1.79)		59/64	1.14 (0.63-2.05)	
		GA/AA	97/91	0.93 (0.59-1.48)	0.77	92/75	1.85 (1.12-3.05)	0.02	181/151	1.02 (0.64-1.63)	0.93	179/159	1.44 (0.87-2.39)	0.16
IL1R1	rs2041748	CC	107/94	1.00 (ref)	0.40	86/84	1.00 (ref)	0.76	210/172	1.00 (ref)	0.81	179/177	1.00 (ref)	0.93
		CT	69/59	0.96 (0.59-1.56)		53/59	0.94 (0.57-1.55)		69/59	0.96 (0.59-1.56)		53/59	0.94 (0.57-1.55)	

		TT	10/13	0.59 (0.23-1.51)		4/4	0.85 (0.19-3.75)		2/1	0.98 (0.08-11.43)		2/3	0.71 (0.11-4.69)	
		CT/TT	79/72	0.89 (0.56-1.41)	0.62	57/63	0.93 (0.57-1.52)	0.78	27/23	1.09 (0.58-2.02)	0.79	37/33	1.06 (0.61-1.84)	0.83
IL1R2	rs2228139	CC	165/146	1.00 (ref)	0.78	132/135	1.00 (ref)	0.90	214/175	1.00 (ref)	0.90	191/190	1.00 (ref)	0.71
		CG	19/17	0.97 (0.46-2.07)		9/10	0.94 (0.35-2.53)		23/19	1.02 (0.53-1.96)		24/20	1.14 (0.58-2.21)	
		GG	1/0			0/0			1/1	0.53 (0.03-9.48)		0/0		
		CG/GG	20/17	1.04 (0.50-2.18)	0.92		n/a		24/20	0.99 (0.52-1.88)	0.97		n/a	
IL1RN	rs4251961	TT	80/63	1.00 (ref)	0.99	55/52	1.00 (ref)	0.22	153/133	1.00 (ref)	0.57	157/133	1.00 (ref)	0.04
		TC	68/72	0.76 (0.45-1.26)		70/75	0.88 (0.52-1.50)		79/57	1.16 (0.75-1.79)		51/66	0.68 (0.43-1.08)	
		CC	32/26	1.15 (0.58-2.27)		12/20	0.56 (0.24-1.30)		7/5	1.03 (0.31-3.48)		6/11	0.44 (0.15-1.31)	
		TC/CC	100/98	0.85 (0.53-1.37)	0.51	82/95	0.81 (0.49-1.35)	0.42	86/62	1.15 (0.75-1.76)	0.51	57/77	0.65 (0.42-1.01)	0.06
IL1RN	rs419598	TT	100/83	1.00 (ref)	0.39	67/83	1.00 (ref)	0.24	184/150	1.00 (ref)	0.75	170/155	1.00 (ref)	0.42
		TC	66/58	0.99 (0.60-1.63)		53/48	1.48 (0.87-2.50)		38/33	0.97 (0.57-1.65)		35/40	0.89 (0.52-1.50)	
		CC	13/17	0.61 (0.27-1.42)		11/12	1.25 (0.50-3.16)		4/1	2.85 (0.29-27.91)		2/5	0.48 (0.09-2.68)	
		TC/CC	79/75	0.90 (0.56-1.43)	0.65	64/60	1.43 (0.87-2.36)	0.16	42/34	1.02 (0.61-1.72)	0.93	37/45	0.84 (0.51-1.41)	0.52
IL1RN	rs454078	AA	101/84	1.00 (ref)	0.51	69/80	1.00 (ref)	0.21	207/166	1.00 (ref)	0.94	188/178	1.00 (ref)	0.51
		AT	72/67	0.95 (0.58-1.53)		61/56	1.28 (0.77-2.12)		29/29	0.84 (0.47-1.50)		28/28	1.06 (0.58-1.93)	
		TT	13/15	0.71 (0.30-1.68)		13/10	1.61 (0.64-4.10)		3/0			0/4		
		AT/TT	85/82	0.90 (0.57-1.43)	0.66	74/66	1.33 (0.82-2.15)	0.25	32/29	0.92 (0.52-1.64)	0.78	28/32	0.93 (0.52-1.67)	0.81
IL1RN	rs579543	CC	104/81	1.00 (ref)	0.26	69/78	1.00 (ref)	0.74	185/156	1.00 (ref)	0.58	170/164	1.00 (ref)	0.72
		CT	68/72	0.70 (0.43-1.15)		64/55	1.30 (0.79-2.16)		51/37	1.16 (0.71-1.92)		43/43	1.14 (0.69-1.88)	
		TT	13/13	0.82 (0.34-1.94)		10/13	0.82 (0.32-2.09)		3/2	1.06 (0.17-6.58)		2/3	0.85 (0.13-5.59)	
		CT/TT	81/85	0.72 (0.46-1.15)	0.17	74/68	1.21 (0.75-1.96)	0.44	54/39	1.16 (0.71-1.88)	0.56	45/46	1.12 (0.68-1.83)	0.66
IL6	rs10499563	TT	120/102	1.00 (ref)	0.16	84/99	1.00 (ref)	0.09	152/119	1.00 (ref)	0.53	137/142	1.00 (ref)	0.23
		TC	63/56	0.91 (0.56-1.48)		51/40	1.46 (0.86-2.48)		77/68	0.84 (0.56-1.28)		66/57	1.31 (0.84-2.05)	
		CC	3/8	0.20 (0.05-0.91)		8/6	1.95 (0.62-6.13)		9/7	0.97 (0.33-2.83)		13/9	1.36 (0.54-3.40)	
		TC/CC	66/64	0.82 (0.51-1.31)	0.40	59/46	1.52 (0.92-2.52)	0.10	86/75	0.86 (0.57-1.28)	0.45	79/66	1.32 (0.86-2.01)	0.20
IL6	rs1800797	GG	82/67	1.00 (ref)	0.33	69/56	1.00 (ref)	0.86	211/166	1.00 (ref)	0.61	190/186	1.00 (ref)	0.94
		GA	93/83	1.04 (0.64-1.67)		50/75	0.57 (0.34-0.96)		26/27	0.80 (0.43-1.49)		22/20	0.94 (0.47-1.87)	
		AA	11/16	0.49 (0.20-1.20)		24/15	1.41 (0.65-3.08)		1/0			1/0		
		GA/AA	104/99	0.93 (0.59-1.48)	0.78	74/90	0.70 (0.43-1.15)	0.16	27/27	0.82 (0.44-1.53)	0.54	23/20	0.98 (0.49-1.95)	0.95
IL6	rs1800796	GG	156/147	1.00 (ref)	0.64	125/122	1.00 (ref)	0.33	184/148	1.00 (ref)	0.86	164/171	1.00 (ref)	0.14
		GC	30/18	1.33 (0.67-2.66)		18/23	0.88 (0.44-1.75)		51/46	0.86 (0.54-1.38)		44/36	1.28 (0.76-2.15)	
		CC	0/1			0/2			4/1	2.70 (0.29-25.12)		7/3	2.51 (0.60-10.46)	

		GC/CC	30/19	1.26 (0.64-2.50)	0.51	18/25	0.79 (0.40-1.56)	0.49	55/47	0.91 (0.57-1.44)	0.67	51/39	1.38 (0.84-2.26)	0.21
IL6	rs1800795	GG	81/67	1.00 (ref)	0.56	67/56	1.00 (ref)	0.99	212/168	1.00 (ref)	0.67	193/188	1.00 (ref)	0.66
		GC	91/77	1.11 (0.69-1.81)		52/75	0.62 (0.37-1.05)		25/26	0.80 (0.43-1.51)		22/22	0.86 (0.44-1.68)	
		CC	14/18	0.62 (0.27-1.42)		23/15	1.44 (0.66-3.15)		2/1	1.64 (0.13-19.93)		0/0	n/a	
		GC/CC	105/95	1.01 (0.63-1.61)	0.97	75/90	0.75 (0.46-1.23)	0.26	27/27	0.84 (0.45-1.55)	0.57		n/a	
IL6R	rs2228145	AA	77/61	1.00 (ref)	0.96	47/56	1.00 (ref)	0.34	186/146	1.00 (ref)	0.27	159/146	1.00 (ref)	0.41
		AC	78/80	0.78 (0.48-1.29)		75/65	1.35 (0.79-2.30)		52/43	0.95 (0.59-1.53)		50/61	0.69 (0.43-1.10)	
		CC	31/24	1.19 (0.59-2.39)		21/24	1.31 (0.63-2.76)		1/6	0.16 (0.02-1.34)		6/3	2.01 (0.45-8.89)	
		AC/CC	109/104	0.87 (0.54-1.38)		96/89	1.34 (0.81-2.23)	0.25	53/49	0.86 (0.54-1.37)		56/64	0.74 (0.47-1.17)	0.20
IL8	rs4073	TT	65/60	1.00 (ref)	0.29	48/46	1.00 (ref)	0.18	6/10	1.00 (ref)	0.70	7/12	1.00 (ref)	0.94
		TA	80/79	0.95 (0.57-1.59)		71/71	0.89 (0.51-1.53)		80/61	2.43 (0.81-7.26)		80/77	1.47 (0.53-4.13)	
		AA	35/22	1.62 (0.80-3.29)		18/30	0.59 (0.28-1.23)		149/124	2.15 (0.73-6.39)		124/120	1.36 (0.49-3.80)	
		TA/AA	115/101	1.09 (0.67-1.77)	0.73	89/101	0.80 (0.48-1.33)	0.38	229/185	2.28 (0.78-6.62)	0.13	204/197	1.41 (0.52-3.86)	0.50
IL8	rs2227306	CC	85/69	1.00 (ref)	0.39	57/58	1.00 (ref)	0.37	178/151	1.00 (ref)	0.61	172/161	1.00 (ref)	0.65
		CT	63/67	0.91 (0.54-1.52)		65/61	1.02 (0.6-01.73)		44/32	1.28 (0.75-2.16)		34/31	0.98 (0.55-1.72)	
		TT	27/19	1.61 (0.76-3.40)		13/23	0.61 (0.28-1.37)		4/4	0.75 (0.17-3.20)		1/3	0.36 (0.03-3.72)	
		CT/TT	90/86	1.05 (0.65-1.70)	0.85	78/84	0.91 (0.55-1.50)	0.72	48/36	1.21 (0.73-2.00)	0.46	35/34	0.93 (0.53-1.63)	0.80
IL16	rs4778889	TT	124/102	1.00 (ref)	0.25	101/101	1.00 (ref)	0.86	77/70	1.00 (ref)	0.38	76/76	1.00 (ref)	0.94
		TC	56/55	0.80 (0.48-1.32)		35/37	0.97 (0.55-1.69)		121/96	1.14 (0.73-1.76)		104/96	1.07 (0.69-1.67)	
		CC	4/6	0.48 (0.09-2.54)		7/8	0.92 (0.30-2.80)		36/28	1.30 (0.70-2.40)		33/37	0.94 (0.52-1.71)	
		TC/CC	60/61	0.77 (0.47-1.26)	0.30	42/45	0.96 (0.57-1.62)	0.87	157/124	1.17 (0.77-1.77)	0.46	137/133	1.04 (0.68-1.58)	0.86
IL17A	rs7747909	GG	101/93	1.00 (ref)	0.25	85/82	1.00 (ref)	0.23	191/151	1.00 (ref)	0.81	184/175	1.00 (ref)	0.55
		GA	73/59	1.44 (0.88-2.35)		52/53	0.95 (0.58-1.58)		41/41	0.83 (0.50-1.37)		32/33	0.96 (0.55-1.68)	
		AA	11/11	1.18 (0.45-3.09)		4/11	0.34 (0.10-1.16)		6/3	1.60 (0.37-6.89)		0/2		
		GA/AA	84/70	1.40 (0.87-2.23)	0.16	56/64	0.85 (0.52-1.39)	0.52	47/44	0.88 (0.55-1.42)	0.61	32/35	0.90 (0.52-1.56)	0.72
IL17A	rs1974226	GG	122/110	1.00 (ref)	0.80	94/91	1.00 (ref)	0.73	186/150	1.00 (ref)	0.82	169/170	1.00 (ref)	0.16
		GA	55/50	1.01 (0.61-1.68)		42/51	0.79 (0.47-1.34)		44/37	1.00 (0.61-1.66)		43/36	1.24 (0.74-2.08)	
		AA	8/6	1.23 (0.38-3.97)		7/5	1.34 (0.39-4.58)		8/6	0.81 (0.26-2.46)		3/0		
		GA/AA	63/56	1.04 (0.64-1.68)	0.88	49/56	0.84 (0.51-1.40)	0.51	52/43	0.97 (0.61-1.56)	0.90	46/36	1.34 (0.80-2.23)	0.26
FGF2	rs308379	AA	78/72	1.00 (ref)	0.33	58/73	1.00 (ref)	0.11	158/134	1.00 (ref)	0.92	142/155	1.00 (ref)	0.18
		AT	78/67	1.32 (0.80-2.17)		64/58	1.37 (0.82-2.30)		73/55	0.99 (0.64-1.55)		63/47	1.36 (0.85-2.16)	
		TT	28/24	1.30 (0.65-2.59)		19/15	1.74 (0.78-3.87)		7/6	0.91 (0.28-2.89)		8/6	1.45 (0.47-4.50)	
		AT/TT	106/91	1.31 (0.82-2.10)	0.26	83/73	1.44 (0.89-2.35)	0.14	80/61	0.99 (0.64-1.52)	0.95	71/53	1.37 (0.88-2.13)	0.17

FGF2	rs1048201	CC	132/113	1.00 (ref)	0.63	89/104	1.00 (ref)	0.12	214/171	1.00 (ref)	0.60	181/191	1.00 (ref)	0.12
		CT	45/42	1.05 (0.62-1.79)		41/37	1.26 (0.73-2.18)		23/22	0.79 (0.41-1.51)		34/18	1.86 (0.98-3.51)	
		TT	8/8	1.35 (0.46-3.96)		11/5	2.35 (0.76-7.27)		2/1	1.33 (0.12-15.23)		0/1		
		CT/TT	53/50	1.09 (0.66-1.81)	0.72	52/42	1.39 (0.83-2.33)	0.21	25/23	0.82 (0.44-1.53)	0.52	34/19	1.75 (0.93-3.28)	0.08
NOD2	rs2066842	CC	100/93	1.00 (ref)	0.70	84/75	1.00 (ref)	0.03	210/177	1.00 (ref)	0.23	190/187	1.00 (ref)	0.47
		CT	71/59	0.91 (0.55-1.49)		48/62	0.60 (0.35-1.01)		28/16	1.75 (0.88-3.49)		26/22	1.36 (0.70-2.62)	
		TT	8/9	0.90 (0.32-2.57)		5/10	0.43 (0.13-1.35)		1/2	0.58 (0.05-7.26)		0/1		
		CT/TT	79/68	0.91 (0.56-1.46)	0.69	53/72	0.57 (0.35-0.96)	0.03	29/18	1.64 (0.84-3.21)	0.15	26/23	1.33 (0.69-2.57)	0.39

[a] OR, odds ratio; 95%CI, 95% confidence interval

[b] Adjusted for age at diagnosis, education, body mass index, family history of breast cancer, history of benign breast disease, smoking status, and proportion of European ancestry.

[c] *P*-trend for genetic dose response determined by coding genotypes as having 0, 1, or 2 variant allele, which was subsequently analyzed as an ordinal variable.

[d] *P* for heterogeneity from dominant models (heterozygous and homozygous variant combined vs. homozygous common).
