# Supplementary material for: Innate Immunity Pathways and Breast Cancer Risk in African American and European-American Women in the Women’s Circle of Health Study (WCHS)
Source: PLoS One. 2013 Aug 21;8(8):e72619. doi: 10.1371/journal.pone.0072619 (PMC3749137; doi:10.1371/journal.pone.0072619)
Supplement: Table S4 — Single nucleotide polymorphisms (SNPs) of innate immune response related pathways and risk of breast cancer by estrogen receptor status among 650 European American and 864 African American women in the WCHS. (PDF) [file pone.0072619.s004.pdf]

Table S4. Single nucleotide polymorphisms (SNPs) of innate immune response related pathways and risk of breast cancer by estrogen receptor status among 650 European American and 864 African American women in the WCHS[a]

Gene	SNP	Genotype	European American						African American					
			Estrogen Receptor positive			Estrogen Receptor negative			Estrogen Receptor positive			Estrogen Receptor negative		
			# Case/ Control	OR (95% CI)[b,c]	*P*[d,e]	#Case/ Control	OR (95% CI)[b,c]	*P*[d,e]	# Case/ Control	OR (95% CI)[b,c]	*P*[d,e]	# Case/ Control	OR (95% CI)[b,c]	*P*[d,e]
TNFA	rs1799964	TT	121/198	1.00 (ref)	0.46	28/198	1.00 (ref)	0.31	149/286	1.00 (ref)	0.12	75/286	1.00 (ref)	0.33
		TC	64/96	1.13 (0.75-1.70)		19/96	1.43 (0.73-2.78)		67/102	1.26 (0.86-1.84)		22/102	0.84 (0.49-1.45)	
		CC	11/13	1.27 (0.52-3.06)		2/13	1.41 (0.28-7.05)		15/16	1.56 (0.73-3.36)		3/16	0.58 (0.16-2.10)	
		TC/CC	75/109	1.15 (0.78-1.69)	0.48	21/109	1.43 (0.75-2.72)	0.28	82/118	1.31 (0.91-1.86)	0.14	25/118	0.80 (0.48-1.34)	0.40
TNFA	rs1800630	CC	140/224	1.00 (ref)	0.38	35/224	1.00 (ref)	0.80	167/320	1.00 (ref)	0.08	83/320	1.00 (ref)	0.43
		CA	50/81	1.04 (0.68-1.60)		13/81	1.05 (0.52-2.15)		57/76	1.47 (0.98-2.21)		15/76	0.80 (0.43-1.49)	
		AA	9/6	2.11 (0.68-6.55)		1/6	1.40 (0.14-13.55)		6/8	1.31 (0.44-3.94)		2/8	0.72 (0.15-3.56)	
		CA/AA	59/87	1.12 (0.74-1.68)	0.60	14/87	1.07 (0.53-2.15)	0.84	63/84	1.46 (0.98-2.15)	0.06	17/84	0.79 (0.44-1.42)	0.43
TNFA	rs1799724	CC	126/231	1.00 (ref)	0.001	31/231	1.00 (ref)	0.11	218/373	1.00 (ref)	0.53	93/373	1.00 (ref)	0.68
		CT	56/66	1.88 (1.21-2.93)		16/66	1.98 (0.98-3.99)		12/30	0.8 (0.39-1.63)		7/30	1.2 (0.49-2.95)	
		TT	9/7	3.17 (1.10-9.12)		1/7	1.12 (0.12-10.58)		0/0			0/0		
		CT/TT	65/73	2.00 (1.31-3.05)	0.001	17/73	1.90 (0.96-3.77)	0.07		n/a			n/a	
TNFA	rs1800629	GG	140/225	1.00 (ref)	0.81	39/225	1.00 (ref)	0.61	190/310	1.00 (ref)	0.09	74/310	1.00 (ref)	0.86
		GA	45/80	0.88 (0.57-1.38)		9/80	0.58 (0.26-1.29)		39/86	0.77 (0.50-1.19)		23/86	1.05 (0.61-1.81)	
		AA	3/3	1.62 (0.31-8.55)		2/3	3.53 (0.53-23.57)		2/9	0.33 (0.07-1.65)		2/9	1.03 (0.21-5.02)	
		GA/AA	48/83	0.91 (0.59-1.41)	0.67	11/83	0.69 (0.33-1.45)	0.33	41/95	0.73 (0.47-1.11)	0.14	25/95	1.05 (0.62-1.78)	0.85
TNFA	rs361525	GG	173/276	1.00 (ref)	0.78	42/276	1.00 (ref)	0.31	209/373	1.00 (ref)	0.58	92/373	1.00 (ref)	0.97
		GA	18/27	0.98 (0.50-1.95)		5/27	1.21 (0.42-3.48)		21/29	1.26 (0.69-2.32)		8/29	1.09 (0.47-2.57)	
		AA	0/1			1/1	9.11 (0.47-176.4)		0/1			0/1		
		GA/AA	18/28	0.95 (0.48-1.86)	0.87	6/28	1.41 (0.52-3.81)	0.50	21/30	1.22 (0.67-2.24)	0.51	8/30	1.05 (0.45-2.46)	0.90
TNFRSF1A	rs767455	TT	62/96	1.00 (ref)	0.90	15/96	1.00 (ref)	0.39	85/177	1.00 (ref)	0.49	36/177	1.00 (ref)	0.06
		TC	98/161	0.92 (0.60-1.41)		22/161	0.88 (0.42-1.84)		123/176	1.44 (1.01-2.06)		46/176	1.41 (0.85-2.32)	
		CC	39/55	1.07 (0.62-1.87)		13/55	1.56 (0.66-3.69)		22/52	0.92 (0.51-1.64)		17/52	1.84 (0.93-3.65)	
		TC/CC	137/216	0.96 (0.64-1.44)	0.84	35/216	1.05 (0.53-2.08)	0.88	145/228	1.32 (0.94-1.87)	0.11	63/228	1.50 (0.94-2.41)	0.09
TNFRSF1A	rs4149570	GG	66/115	1.00 (ref)	0.19	22/115	1.00 (ref)	0.76	177/314	1.00 (ref)	0.73	79/314	1.00 (ref)	0.61
		GT	104/154	1.27 (0.84-1.92)		20/154	0.67 (0.34-1.34)		49/86	0.97 (0.64-1.48)		17/86	0.81 (0.45-1.47)	
		TT	29/42	1.42 (0.78-2.56)		8/42	1.06 (0.42-2.68)		5/5	1.96 (0.52-7.30)		4/5	4.53 (1.08-19.09)	
		GT/TT	133/196	1.30 (0.87-1.93)	0.20	28/196	0.75 (0.40-1.43)	0.38	54/91	1.01 (0.67-1.53)	0.95	21/91	0.96 (0.55-1.68)	0.89
CCL2	rs1024611	TT	96/171	1.00 (ref)	0.54	26/171	1.00 (ref)	0.59	143/268	1.00 (ref)	0.42	68/268	1.00 (ref)	0.47
		TC	90/116	1.35 (0.91-1.99)		22/116	1.1 (0.57-2.12)		83/123	1.28 (0.89-1.83)		32/123	1.08 (0.66-1.75)	

		CC	11/22	0.79 (0.35-1.79)		1/22	0.25 (0.03-2.51)		5/14	0.76 (0.26-2.21)		0/14		
		TC/CC	101/138	1.26 (0.86-1.83)	0.23	23/138	0.99 (0.52-1.90)	0.98	88/137	1.23 (0.87-1.74)	0.25	32/137	0.96 (0.60-1.56)	0.88
CCL2	rs2857656	GG	91/166	1.00 (ref)	0.54	26/166	1.00 (ref)	0.52	74/154	1.00 (ref)	0.10	40/154	1.00 (ref)	0.63
		GC	85/112	1.35 (0.90-2.01)		21/112	1.03 (0.53-2.03)		108/187	1.16 (0.80-1.70)		45/187	0.94 (0.57-1.53)	
		CC	10/21	0.79 (0.33-1.85)		1/21	0.25 (0.02-2.61)		44/60	1.54 (0.94-2.54)		13/60	0.84 (0.41-1.71)	
		GC/CC	95/133	1.26 (0.86-1.86)	0.24	22/133	0.94 (0.48-1.83)	0.85	152/247	1.25 (0.88-1.79)	0.22	58/247	0.91 (0.57-1.45)	0.70
CCL2	rs4586	TT	64/141	1.00 (ref)	0.19	19/141	1.00 (ref)	0.74	21/45	1.00 (ref)	0.01	15/45	1.00 (ref)	0.30
		TC	99/128	1.67 (1.10-2.55)		26/128	1.46 (0.74-2.86)		96/203	1.13 (0.62-2.06)		45/203	0.65 (0.32-1.32)	
		CC	21/37	1.11 (0.57-2.15)		5/37	0.84 (0.25-2.81)		112/156	1.74 (0.95-3.20)		37/156	0.62 (0.30-1.29)	
		TC/CC	120/165	1.55 (1.03-2.31)	0.03	31/165	1.34 (0.70-2.59)	0.38	208/359	1.37 (0.77-2.44)	0.28	82/359	0.64 (0.33-1.25)	0.19
CCL2	rs13900	CC	96/171	1.00 (ref)	0.46	26/171	1.00 (ref)	0.63	143/266	1.00 (ref)	0.45	68/266	1.00 (ref)	0.46
		CT	90/117	1.34 (0.91-1.97)		22/117	1.09 (0.56-2.10)		82/123	1.27 (0.88-1.81)		32/123	1.07 (0.66-1.75)	
		TT	11/20	0.86 (0.37-1.97)		1/20	0.27 (0.03-2.74)		5/14	0.75 (0.26-2.19)		0/14		
		CT/TT	101/137	1.27 (0.87-1.85)	0.22	23/137	0.99 (0.52-1.91)	0.98	87/137	1.22 (0.86-1.73)	0.27	32/137	0.96 (0.59-1.56)	0.87
CCL5	rs3817655	TT	128/202	1.00 (ref)	0.70	32/202	1.00 (ref)	0.99	73/131	1.00 (ref)	0.72	41/131	1.00 (ref)	0.03
		TA	54/98	0.84 (0.54-1.29)		18/98	1.24 (0.64-2.41)		105/198	0.90 (0.62-1.33)		43/198	0.62 (0.38-1.03)	
		AA	7/8	1.28 (0.43-3.82)		0/8			53/76	1.13 (0.70-1.81)		16/76	0.52 (0.27-1.02)	
		TA/AA	61/106	0.87 (0.58-1.32)	0.52	18/106	1.13 (0.58-2.19)	0.71	158/274	0.97 (0.67-1.38)	0.85		0.59 (0.37-0.95)	0.03
CCL5	rs2280789	TT	140/236	1.00 (ref)	0.38	37/236	1.00 (ref)	0.85	136/267	1.00 (ref)	0.04	71/267	1.00 (ref)	0.11
		TC	56/72	1.25 (0.81-1.94)		13/72	1.23 (0.60-2.52)		80/114	1.47 (1.02-2.12)		29/114	0.89 (0.54-1.47)	
		CC	3/5	0.98 (0.21-4.54)		0/5			13/17	1.56 (0.71-3.42)		0/17		
		TC/CC	59/77	1.24 (0.81-1.89)	0.33	13/77	1.16 (0.56-2.37)	0.69	93/131	1.48 (1.04-2.11)	0.03	29/131	0.77 (0.47-1.27)	0.31
CCL5	rs2107538	CC	126/200	1.00 (ref)	0.96	30/200	1.00 (ref)	0.70	71/131	1.00 (ref)	0.46	40/131	1.00 (ref)	0.02
		CT	58/94	0.99 (0.65-1.51)		18/94	1.47 (0.75-2.87)		106/199	0.93 (0.63-1.37)		47/199	0.67 (0.41-1.11)	
		TT	6/9	0.99 (0.32-3.02)		0/9			53/73	1.24 (0.77-2.00)		13/73	0.45 (0.22-0.92)	
		CT/TT	64/103	0.99 (0.66-1.49)	0.96	18/103	1.32 (0.68-2.58)	0.41	159/272	1.01 (0.70-1.45)	0.95	60/272	0.61 (0.38-0.98)	0.04
CXCL5	rs425535	GG	144/240	1.00 (ref)	0.61	39/240	1.00 (ref)	0.72	94/142	1.00 (ref)	0.16	34/142	1.00 (ref)	0.51
		GA	40/63	1.01 (0.63-1.62)		11/63	1.01 (0.47-2.18)		106/198	0.78 (0.54-1.12)		54/198	1.10 (0.67-1.82)	
		AA	6/5	1.72 (0.48-6.15)		0/5			31/65	0.73 (0.44-1.23)		12/65	0.70 (0.33-1.47)	
		GA/AA	46/68	1.07 (0.68-1.68)	0.78	11/68	0.94 (0.44-2.02)	0.88	137/263	0.77 (0.54-1.09)	0.14	66/263	1.00 (0.62-1.62)	1.00
CRP	rs1205	CC	82/118	1.00 (ref)	0.41	24/118	1.00 (ref)	0.64	146/257	1.00 (ref)	0.80	60/257	1.00 (ref)	0.98
		CT	88/149	0.89 (0.59-1.35)		17/149	0.60 (0.30-1.20)		72/121	1.07 (0.74-1.55)		37/121	1.31 (0.81-2.11)	
		TT	19/35	0.77 (0.40-1.49)		7/35	1.12 (0.42-2.95)		12/25	0.76 (0.36-1.60)		3/25	0.50 (0.14-1.74)	
		CT/TT	107/184	0.87 (0.59-1.29)	0.48	24/184	0.69 (0.36-1.31)	0.26	84/146	1.01 (0.71-1.44)	0.94	40/146	1.16 (0.73-1.85)	0.52
CRP	rs1130864	CC	88/157	1.00 (ref)	0.20	26/157	1.00 (ref)	0.47	147/266	1.00 (ref)	0.44	60/266	1.00 (ref)	0.77
		CT	82/118	1.28 (0.85-1.91)		14/118	0.69 (0.33-1.44)		53/89	1.02 (0.68-1.55)		23/89	1.22 (0.70-2.13)	

		TT	20/24	1.38 (0.69-2.75)		8/24	2.19 (0.84-5.71)		5/18	0.50 (0.18-1.40)		3/18	0.83 (0.23-3.01)	
		CT/TT	102/142	1.29 (0.88-1.90)	0.19	22/142	0.93 (0.49-1.77)	0.83	58/107	0.94 (0.63-1.39)	0.74	26/107	1.15 (0.68-1.96)	0.60
CRP	rs1800947	GG	167/262	1.00 (ref)	0.90	40/262	1.00 (ref)	0.34	227/392	1.00 (ref)	0.14	100/392	1.00 (ref)	0.99
		GC	23/40	0.97 (0.54-1.73)		8/40	1.67 (0.70-4.01)		3/11	0.37 (0.10-1.40)		0/11		
		CC	1/2	0.92 (0.08-11.08)		0/2			0/0			0/0		
		GC/CC	24/42	0.97 (0.55-1.71)	0.91	8/42	1.61 (0.68-3.85)	0.28		n/a				
IL1A	rs17561	GG	95/154	1.00 (ref)	0.80	26/154	1.00 (ref)	0.79	161/287	1.00 (ref)	0.83	71/287	1.00 (ref)	0.62
		GT	83/118	1.30 (0.87-1.95)		18/118	1.14 (0.57-2.27)		62/108	0.99 (0.67-1.45)		29/108	1.12 (0.67-1.86)	
		TT	21/41	0.88 (0.48-1.63)		5/41	0.75 (0.26-2.17)		7/10	1.29 (0.47-3.55)		0/10		
		GT/TT	104/159	1.19 (0.82-1.74)	0.36	23/159	1.02 (0.54-1.95)	0.94	69/118	1.01 (0.70-1.47)	0.94	29/118	1.00 (0.61-1.66)	0.99
IL1A	rs1800587	CC	92/153	1.00 (ref)	0.78	26/153	1.00 (ref)	0.95	86/155	1.00 (ref)	0.99	37/155	1.00 (ref)	0.78
		CT	84/116	1.30 (0.87-1.95)		17/116	1.04 (0.52-2.10)		109/186	1.06 (0.74-1.54)		48/186	1.03 (0.63-1.69)	
		TT	20/39	0.88 (0.47-1.64)		6/39	0.93 (0.34-2.55)		35/64	0.97 (0.59-1.62)		15/64	0.88 (0.44-1.75)	
		CT/TT	104/155	1.19 (0.82-1.74)	0.36	23/155	1.01 (0.53-1.92)	0.97	144/250	1.04 (0.73-1.47)	0.82	63/250	0.99 (0.62-1.58)	0.96
IL1B	rs1143634	CC	108/180	1.00 (ref)	0.71	30/180	1.00 (ref)	0.98	172/306	1.00 (ref)	0.33	77/306	1.00 (ref)	0.99
		CT	76/103	1.32 (0.89-1.97)		15/103	1.06 (0.53-2.15)		52/96	0.97 (0.65-1.45)		21/96	0.86 (0.50-1.49)	
		TT	13/26	0.80 (0.38-1.66)		4/26	0.91 (0.28-2.91)		7/3	4.74 (1.16-19.3)		2/3	3.12 (0.46-21.05)	
		CT/TT	89/129	1.21 (0.83-1.76)	0.33	19/129	1.03 (0.53-1.97)	0.94	59/99	1.08 (0.73-1.59)	0.71	23/99	0.92 (0.54-1.57)	0.76
IL1B	rs1143627	TT	83/145	1.00 (ref)	0.19	28/145	1.00 (ref)	0.22	42/74	1.00 (ref)	0.65	15/74	1.00 (ref)	0.79
		TC	83/134	1.11 (0.74-1.66)		17/134	0.65 (0.33-1.29)		110/178	1.11 (0.70-1.76)		52/178	1.58 (0.82-3.04)	
		CC	32/33	1.52 (0.84-2.73)		5/33	0.63 (0.21-1.87)		79/153	0.93 (0.58-1.52)		33/153	1.06 (0.53-2.11)	
		TC/CC	115/167	1.20 (0.82-1.74)	0.35	22/167	0.64 (0.34-1.22)	0.18	189/331	1.03 (0.67-1.59)	0.89	85/331	1.33 (0.72-2.47)	0.36
IL1B	rs16944	GG	83/143	1.00 (ref)	0.22	27/143	1.00 (ref)	0.27	47/93	1.00 (ref)	0.92	21/93	1.00 (ref)	0.67
		GA	83/134	1.08 (0.72-1.61)		17/134	0.67 (0.33-1.33)		118/185	1.24 (0.80-1.92)		52/185	1.33 (0.74-2.38)	
		AA	31/32	1.52 (0.84-2.75)		5/32	0.67 (0.22-2.01)		66/125	1.05 (0.65-1.70)		26/125	0.90 (0.47-1.74)	
		GA/AA	114/166	1.17 (0.8-1.70)	0.43	22/166	0.67 (0.35-1.27)	0.22	184/310	1.17 (0.78-1.76)	0.46	78/310	1.15 (0.67-2.00)	0.61
IL1R1	rs2041748	CC	112/178	1.00 (ref)	0.98	34/178	1.00 (ref)	0.12	197/349	1.00 (ref)	0.68	86/349	1.00 (ref)	1.00
		CT	79/118	1.12 (0.76-1.65)		15/118	0.66 (0.34-1.31)		31/52	1.18 (0.71-1.95)		12/52	1.03 (0.51-2.07)	
		TT	8/17	0.71 (0.28-1.80)		1/17	0.31 (0.04-2.52)		2/4	0.75 (0.12-4.71)		1/4	0.85 (0.09-8.34)	
		CT/TT	87/135	1.06 (0.73-1.55)	0.75	16/135	0.62 (0.32-1.20)		33/56	1.15 (0.70-1.88)	0.58	13/56	1.02 (0.52-2.00)	
IL1R2	rs2228139	CC	185/281	1.00 (ref)	0.23	41/281	1.00 (ref)	0.12	202/365	1.00 (ref)	0.60	93/365	1.00 (ref)	0.20
		CG	12/27	0.64 (0.31-1.33)		7/27	1.48 (0.57-3.87)		28/39	1.16 (0.68-1.98)		6/39	0.60 (0.24-1.48)	
		GG	0/0			1/0			1/1	0.99 (0.06-16.76)		0/1		
		CG/GG		n/a		8/27	1.74 (0.70-4.34)		29/40	1.16 (0.68-1.96)	0.58	6/40	0.57 (0.23-1.41)	
IL1RN	rs4251961	TT	74/115	1.00 (ref)	0.80	22/115	1.00 (ref)	0.95	160/266	1.00 (ref)	0.30	63/266	1.00 (ref)	0.73
		TC	88/147	0.95 (0.63-1.45)		19/147	0.68 (0.34-1.36)		61/123	0.77 (0.53-1.13)		34/123	1.27 (0.78-2.07)	

		CC	27/46	0.94 (0.52-1.69)		9/46	1.18 (0.48-2.92)		9/16	0.95 (0.39-2.28)		2/16	0.58 (0.13-2.66)	
		TC/CC	115/193	0.95 (0.64-1.41)	0.80	28/193	0.78 (0.41-1.49)	0.46	70/139	0.79 (0.55-1.14)	0.21	36/139	1.19 (0.74-1.93)	0.47
IL1RN	rs419598	TT	103/166	1.00 (ref)	0.96	25/166	1.00 (ref)	0.70	178/305	1.00 (ref)	0.99	79/305	1.00 (ref)	0.48
		TC	67/106	1.15 (0.76-1.74)		21/106	1.23 (0.63-2.38)		38/73	0.91 (0.58-1.43)		16/73	0.90 (0.49-1.67)	
		CC	14/29	0.85 (0.42-1.73)		2/29	0.45 (0.10-2.06)		5/6	1.51 (0.43-5.35)		0/6		
		TC/CC	81/135	1.09 (0.74-1.60)	0.67	23/135	1.06 (0.56-2.00)	0.86	43/79	0.96 (0.62-1.47)	0.84	16/79	0.85 (0.46-1.56)	0.60
IL1RN	rs454078	AA	105/164	1.00 (ref)	0.76	26/164	1.00 (ref)	0.62	198/344	1.00 (ref)	0.79	87/344	1.00 (ref)	0.67
		AT	78/123	1.07 (0.72-1.58)		22/123	1.05 (0.55-2.00)		31/57	0.93 (0.56-1.53)		13/57	0.97 (0.49-1.90)	
		TT	16/25	1.07 (0.53-2.14)		2/25	0.50 (0.11-2.31)		2/4	0.97 (0.16-5.81)		0/4		
		AT/TT	94/148	1.07 (0.73-1.55)	0.73	24/148	0.96 (0.51-1.79)	0.89	33/61	0.93 (0.57-1.51)	0.78	13/61	0.91 (0.47-1.78)	0.79
IL1RN	rs579543	CC	108/159	1.00 (ref)	0.48	26/159	1.00 (ref)	0.34	175/320	1.00 (ref)	0.30	77/320	1.00 (ref)	0.95
		CT	75/127	0.89 (0.60-1.32)		22/127	0.95 (0.50-1.81)		52/80	1.24 (0.82-1.88)		22/80	1.17 (0.67-2.05)	
		TT	15/26	0.83 (0.41-1.69)		2/26	0.39 (0.08-1.80)		4/5	1.25 (0.32-4.89)		0/5		
		CT/TT	90/153	0.88 (0.60-1.28)	0.49	24/153	0.84 (0.45-1.58)	0.59	56/85	1.25 (0.83-1.86)	0.28	22/85	1.10 (0.63-1.91)	0.74
IL6R	rs2228145	AA	79/117	1.00 (ref)	0.94	11/117	1.00 (ref)	0.06	170/292	1.00 (ref)	0.83	75/292	1.00 (ref)	0.53
		AC	87/145	0.84 (0.56-1.27)		30/145	1.95 (0.91-4.19)		56/104	0.92 (0.62-1.37)		23/104	0.92 (0.53-1.59)	
		CC	33/48	1.13 (0.65-1.99)		9/48	2.39 (0.88-6.47)		5/9	1.17 (0.36-3.75)		1/9	0.45 (0.05-3.72)	
		AC/CC	120/193	0.91 (0.62-1.33)	0.62	39/193	2.04 (0.98-4.26)	0.06	61/113	0.94 (0.64-1.38)	0.74	24/113	0.88 (0.51-1.51)	0.64
IL6	rs10499563	TT	130/201	1.00 (ref)	0.45	30/201	1.00 (ref)	0.88	152/261	1.00 (ref)	1.00	59/261	1.00 (ref)	0.30
		TC	65/96	1.03 (0.68-1.54)		17/96	0.99 (0.50-1.94)		67/125	0.96 (0.66-1.40)		36/125	1.29 (0.79-2.10)	
		CC	4/14	0.41 (0.12-1.33)		3/14	1.23 (0.31-4.91)		11/16	1.10 (0.48-2.50)		5/16	1.33 (0.46-3.89)	
		TC/CC	69/110	0.94 (0.64-1.40)	0.78	20/110	1.02 (0.53-1.94)	0.96	78/141	0.98 (0.69-1.40)	0.91	41/141	1.30 (0.81-2.06)	0.28
IL6	rs1800797	GG	88/123	1.00 (ref)	0.51	23/123	1.00 (ref)	0.99	201/352	1.00 (ref)	0.93	89/352	1.00 (ref)	0.79
		GA	92/158	0.89 (0.60-1.33)		20/158	0.72 (0.36-1.41)		25/47	0.85 (0.49-1.48)		11/47	1.11 (0.53-2.31)	
		AA	19/31	0.84 (0.43-1.64)		7/31	1.34 (0.50-3.59)		2/0			0/0		
		GA/AA	111/189	0.88 (0.60-1.29)	0.52	27/189	0.82 (0.43-1.54)	0.53	27/47	0.91 (0.53-1.56)	0.72		n/a	
IL6	rs1800796	GG	169/269	1.00 (ref)	0.96	47/269	1.00 (ref)	0.10	176/319	1.00 (ref)	0.78	76/319	1.00 (ref)	0.24
		GC	30/41	1.20 (0.70-2.05)		3/41	0.40 (0.11-1.40)		52/82	1.09 (0.73-1.64)		21/82	1.17 (0.67-2.04)	
		CC	0/3			0/3			2/4	0.8 (0.13-4.82)		3/4	3.32 (0.68-16.24)	
		GC/CC	30/44	1.11 (0.65-1.89)	0.70	3/44	0.36 (0.10-1.26)	0.11	54/86	1.08 (0.72-1.61)	0.72	24/86	1.27 (0.74-2.17)	0.38
IL6	rs1800795	GG	88/123	1.00 (ref)	0.56	20/123	1.00 (ref)	0.29	204/356	1.00 (ref)	0.64	89/356	1.00 (ref)	0.92
		GC	91/152	0.92 (0.62-1.38)		21/152	0.94 (0.46-1.89)		24/48	0.79 (0.46-1.38)		11/48	1.09 (0.53-2.27)	
		CC	19/33	0.83 (0.43-1.62)		9/33	1.97 (0.78-5.01)		2/1	2.56 (0.20-33.38)		0/1		
		GC/CC	110/185	0.90 (0.62-1.33)	0.61	30/185	1.11 (0.58-2.14)	0.74	26/49	0.83 (0.48-1.43)	0.50	11/49	1.07 (0.52-2.21)	0.86
IL8	rs4073	TT	67/106	1.00 (ref)	0.80	17/106	1.00 (ref)	0.81	6/22	1.00 (ref)	0.51	3/22	1.00 (ref)	0.73
		TA	92/150	0.95 (0.62-1.45)		25/150	1.01 (0.50-2.03)		81/138	1.99 (0.76-5.24)		32/138	1.61 (0.44-5.93)	

		AA	30/52	0.94 (0.53-1.66)		8/52	1.15 (0.45-2.95)		141/244	1.93 (0.74-5.07)		61/244	1.58 (0.43-5.72)	
		TA/AA	122/202	0.95 (0.63-1.41)	0.79	33/202	1.04 (0.54-2.02)	0.91	222/382	1.96 (0.76-5.06)	0.16	93/382	1.59 (0.45-5.67)	0.47
IL8	rs2227306	CC	86/127	1.00 (ref)	0.49	20/127	1.00 (ref)	0.59	175/312	1.00 (ref)	1.00	78/312	1.00 (ref)	0.76
		CT	77/128	0.92 (0.60-1.39)		23/128	1.29 (0.64-2.61)		41/63	1.11 (0.70-1.75)		16/63	1.22 (0.65-2.29)	
		TT	20/42	0.81 (0.43-1.52)		6/42	1.18 (0.42-3.32)		2/7	0.54 (0.11-2.75)		1/7	0.68 (0.08-5.89)	
		CT/TT	97/170	0.89 (0.6-1.32)	0.56	29/170	1.27 (0.65-2.47)	0.48	43/70	1.06 (0.68-1.65)	0.81	17/70	1.16 (0.63-2.15)	0.63
IL16	rs4778889	TT	141/203	1.00 (ref)	0.10	31/203	1.00 (ref)	1.00	83/146	1.00 (ref)	0.92	34/146	1.00 (ref)	0.69
		TC	52/92	0.76 (0.49-1.16)		19/92	1.38 (0.71-2.65)		111/192	1.02 (0.71-1.48)		50/192	1.10 (0.67-1.82)	
		CC	5/14	0.48 (0.15-1.57)		0/14			32/65	1.02 (0.60-1.72)		16/65	1.13 (0.57-2.23)	
		TC/CC	57/106	0.72 (0.48-1.10)	0.13	19/106	1.20 (0.63-2.31)	0.57	143/257	1.02 (0.72-1.45)	0.90	66/257	1.11 (0.69-1.79)	0.67
IL17A	rs7747909	GG	117/175	1.00 (ref)	0.54	25/175	1.00 (ref)	0.23	190/326	1.00 (ref)	0.64	86/326	1.00 (ref)	0.23
		GA	74/112	1.08 (0.73-1.60)		20/112	1.45 (0.74-2.85)		37/74	0.82 (0.52-1.28)		12/74	0.63 (0.32-1.24)	
		AA	6/22	0.51 (0.20-1.32)		4/22	1.67 (0.49-5.67)		4/5	1.48 (0.38-5.80)		1/5	0.94 (0.09-9.27)	
		GA/AA	80/134	0.99 (0.68-1.45)	0.96	24/134	1.48 (0.78-2.82)	0.23	41/79	0.86 (0.55-1.32)	0.48	13/79	0.65 (0.34-1.25)	0.19
IL17A	rs1974226	GG	124/201	1.00 (ref)	0.56	36/201	1.00 (ref)	0.25	176/320	1.00 (ref)	0.25	84/320	1.00 (ref)	0.45
		GA	64/101	1.05 (0.71-1.58)		14/101	0.81 (0.41-1.61)		47/73	1.23 (0.81-1.88)		15/73	0.86 (0.46-1.61)	
		AA	10/11	1.37 (0.54-3.44)		0/11			7/6	1.48 (0.47-4.67)		1/6	0.46 (0.05-4.06)	
		GA/AA	74/112	1.09 (0.74-1.60)		14/112	0.74 (0.37-1.46)	0.38	54/79	1.26 (0.84-1.89)		16/79	0.82 (0.45-1.51)	0.53
FGF2	rs308379	AA	79/145	1.00 (ref)	0.05	22/145	1.00 (ref)	0.84	158/289	1.00 (ref)	0.73	65/289	1.00 (ref)	0.32
		AT	86/125	1.33 (0.88-2.00)		21/125	1.12 (0.57-2.21)		63/102	1.07 (0.73-1.58)		31/102	1.35 (0.82-2.23)	
		TT	32/39	1.70 (0.96-3.02)		6/39	1.04 (0.38-2.88)		8/12	1.08 (0.42-2.79)		3/12	1.14 (0.30-4.31)	
		AT/TT	118/164	1.41 (0.96-2.07)	0.08	27/164	1.10 (0.58-2.08)	0.76	71/114	1.07 (0.74-1.56)	0.72	34/114	1.33 (0.82-2.17)	0.25
FGF2	rs1048201	CC	130/217	1.00 (ref)	0.14	40/217	1.00 (ref)	0.25	197/362	1.00 (ref)	0.17	89/362	1.00 (ref)	0.84
		CT	54/79	1.16 (0.76-1.78)		7/79	0.50 (0.21-1.21)		32/40	1.42 (0.85-2.38)		11/40	1.06 (0.51-2.19)	
		TT	13/13	1.90 (0.83-4.36)		2/13	0.91 (0.19-4.45)		2/2	1.59 (0.22-11.77)		0/2		
		CT/TT	67/92	1.26 (0.85-1.88)	0.25	9/92	0.56 (0.25-1.24)	0.15	34/42	1.43 (0.86-2.36)	0.16	11/42	0.99 (0.48-2.05)	0.98
NOD2	rs2066842	CC	104/168	1.00 (ref)	0.40	35/168	1.00 (ref)	0.02	200/364	1.00 (ref)	0.23	88/364	1.00 (ref)	0.36
		CT	74/121	0.83 (0.55-1.25)		14/121	0.45 (0.22-0.92)		31/38	1.60 (0.94-2.72)		12/38	1.56 (0.75-3.25)	
		TT	10/19	0.83 (0.36-1.93)		1/19	0.25 (0.03-1.98)		0/3			0/3		
		CT/TT	84/140	0.83 (0.56-1.23)	0.36	15/140	0.42 (0.21-0.84)	0.02	31/41	1.50 (0.89-2.54)	0.13	12/41	1.5 (0.72-3.11)	0.28

[a] Based on from 254 EA (75.8%) and 332 (72.5%) AA cases with available data on ER status.

[b] OR, odds ratio; 95%CI, 95% confidence interval

[c] Adjusted for age at diagnosis, education, body mass index, family history of breast cancer, history of benign breast disease, menopausal status, smoking status, and proportion of European ancestry.

[d] *P*-trend for genetic dose response determined by coding genotypes as having 0, 1, or 2 variant allele, which was subsequently analyzed as an ordinal variable.

[e] *P* for heterogeneity from dominant models (heterozygous and homozygous variant combined vs. homozygous common).
